# Supplementary material for: Virulence genes, antimicrobial resistance profile, phylotyping and pathotyping of diarrheagenic Escherichia coli isolated from children in Southwest Mexico
Source: PLoS One. 2024 Mar 12;19(3):e0300304. doi: 10.1371/journal.pone.0300304 (PMC10931464; doi:10.1371/journal.pone.0300304)
Supplement: S1 Table — (DOCX) [file pone.0300304.s001.docx]

| **Supplementary table 1. *Escherichia coli* pathotypes and associated virulence genes** | | | | | | |
| --- | --- | --- | --- | --- | --- | --- |
| **Pathotype** | **Location of essential virulence determinant(s)** | **Adhesion/**  **invasion**  **gene** | **Function** | **Toxin**  **gene** | **Function** | **Reference** |
| tEPEC | Pathogenicity island (LEE PAI) | *eaeA* | Intimin |  |  | (1) |
|  | Plasmid (pEAF) | *bfpA* | Type IV bundle-forming pili |  |  |  |
| aEPEC | Pathogenicity island (LEE PAI | *eaeA* | Intimin |  |  | (1) |
| EHEC/STEC | Prophages |  |  | *stx1* | Shiga toxin I | (2) |
|  |  |  |  | *stx2* | Shiga toxin II |  |
|  | Pathogenicity island (LEE PAI | *eaeA* | Intimin |  |  |  |
| ETEC | Plasmid, transposon |  |  | *lt1* | Heat-labile toxin 1 | (3, 4) |
|  |  |  |  | *st1* | Heat-stable toxin 1 |  |
| EAEC | Plasmid (pAA) | *aggR* | Aggregative adhesion |  |  | (5, 6) |
| DAEC | Not know | *afa or dr* | Afimbrial or fimbrial adhesins |  |  | (2) |
| EIEC | Plasmid (pINV) | *ipaH* | Invasion |  |  | (7) |
|  | Plasmid | virF |  |  |  |  |
| *tEPEC, typical enteropathogenic E. coli, LEE PAI, locus of enterocyte effacement pathogenicity island, bfpA, gene for a structural protein of bundle-forming pili, eaeA, gene intimin for intimate adherence, aEPEC, atypical enteropathogenic E. coli, EHEC, enterohemorrhagic E. coli, STEC, shiga toxin-producing E. coli, ETEC, enterotoxigenic E. coli, EAEC, enteroaggregative E. coli, pAA, virulence plasmid of enteroagreggative, aggR, gene for a transcriptional regulator, DAEC, diffusely-adherent E. coli, afa, afimbrial adhesin, dr, fimbrial adhesin, EIEC, enteroinvasive E. coli, pINV, virulence plasmid of enteroinvasive E. coli and Shigella.* | | | | | | |

**References**

1. Trabulsi LR, Keller R, Tardelli Gomes TA. Typical and atypical enteropathogenic Escherichia coli. Emerg Infect Dis. 2002;8(5):508-13.

2. Clements A, Young JC, Constantinou N, Frankel G. Infection strategies of enteric pathogenic Escherichia coli. Gut Microbes. 2012;3(2):71-87.

3. Robins-Browne RM, Holt KE, Ingle DJ, Hocking DM, Yang J, Tauschek M. Are Escherichia coli Pathotypes Still Relevant in the Era of Whole-Genome Sequencing? Front Cell Infect Microbiol. 2016;6:141.

4. Nataro JP, Kaper JB. Diarrheagenic Escherichia coli. Clin Microbiol Rev. 1998;11(1):142-201.

5. Jenkins C, Chart H, Willshaw GA, Cheasty T, Smith HR. Genotyping of enteroaggregative Escherichia coli and identification of target genes for the detection of both typical and atypical strains. Diagn Microbiol Infect Dis. 2006;55(1):13-9.

6. Aslani MM, Alikhani MY, Zavari A, Yousefi R, Zamani AR. Characterization of enteroaggregative Escherichia coli (EAEC) clinical isolates and their antibiotic resistance pattern. Int J Infect Dis. 2011;15(2):e136-9.

7. Adler B, Sasakawa C, Tobe T, Makino S, Komatsu K, Yoshikawa M. A dual transcriptional activation system for the 230 kb plasmid genes coding for virulence-associated antigens of Shigella flexneri. Mol Microbiol. 1989;3(5):627-35.
