# Supplementary material for: Virulence genes, antimicrobial resistance profile, phylotyping and pathotyping of diarrheagenic Escherichia coli isolated from children in Southwest Mexico
Source: PLoS One. 2024 Mar 12;19(3):e0300304. doi: 10.1371/journal.pone.0300304 (PMC10931464; doi:10.1371/journal.pone.0300304)
Supplement: S2 Table — (DOCX) [file pone.0300304.s002.docx]

| Supplementary table 2. Primers used in this study. | | | | | |
| --- | --- | --- | --- | --- | --- |
| Strain/  Pathotype | **Gen** | **Sequence**  **(5´-3´)** | **Size**  **(bp)** | ***Tm* of PCR**  **(°C)** | **Reference** |
| EPEC | *eae* | *f*-TCAATGCAGTTCCGTTATCAGTT  *r*-GTAAAGTCCGTTACCCCAACCTG | 482 | 60 | (1) |
| EPEC | *bfp* | *f*-GGAAGTCAAATTCATGGGGGTAT  *r*-GGAATCAGACGCAGACTGGTAGT | 300 | 60 | (2) |
| EHEC | *stx1* | *f*-CAAAGACGTATGTAGATTCGC  *r*-TTCGTTCAACAATAAGCCGTA | 192 | 60 | (2) |
| EHEC | *stx2* | *f*-TATTATTTAAATGGGTACTGTGC  *r*-CATAACTTTGTTGGGTCGAAA | 96 | 60 | (2) |
| EIEC | *virF* | *f*-AGCTCAGGCAATGAAACTTTGAC  *r*-TGGGCTTGATATTCCGATAAGTC | 618 | 60 | (3) |
| EIEC | *ipaH* | *f*-CTCGGCACGTTTTAATAGTCTGG  *r*-GTGGAGAGCTGAAGTTTCTCTGC | 933 | 60 | (3) |
| EAEC | *aafll* | *f*-CACAGGCAACTGAAATAAGTCTGG  *r*-ATTCCCATGATGTCAAGCACTTC | 378 | 60 | (3) |
| ETEC | *lt* | *f*-GCACACGGAGCTCCTCSAGTCTCC  *r*-TTCATCCTTTCAATGGCTTT | 218 | 60 | (3) |
| DAEC | *daaE* | *f*-GAACGTTGGTTAATGTGGGGTAA  *r*-TATTCACCGGTCGGTTATCAGT | 542 | 60 | (3) |
| EHEC | *ehaC* | *f*-TAATGACGGCAAAGGTGGT  *r*-CATTCATCAGGGAGTTGCT | 599 | 60 | (4) |
| EHEC | *ehaD* | *f*-GGCAGTTGACACGATTATTA  *r*-CTGTCGCTTTGCCATTATC | 821 | 60 | (4) |
| EHEC | *cah* | *f*-CGTATCGCTGTGCCCGGATAAC  *r*-CCGTATACGAGTTGTCAGAATCA | 707 | 58 | (4) |
| EPEC | *nleB* | *f*-TCGCCATCAACAAAAATACC  *r*-GCTTTCACCGATAAGGACAAC | 273 | 55 | (4) |
| EAEC | *pet* | *f*-GGCACAGAATAAAGGGGTGTTT  *r*-CCTCTTGTTTCCACGACATAC | 302 | 58 | (5) |
| EPEC | *espC* | *f*-TAGTGCAGTGCAGAAAGCAGTT  *r*-AGTTTTCCTGTTGCTGTATGCC | 301 | 58 | (5) |
| DAEC | *sat* | *f-*TCAGAAGCTCAGCGAATCATTG  *r*-CCATTATCACCAGTAAAACGCACC | 930 | 58 | (6) |
| EHEC | *hlyA* | *f*-AGCTGCAAGTGCGGGTCTG  *r*-TACGGGTTATGCCTGCAAGTTCAC | 569 | 60 | (2) |
| EAEC | *pic* | *f*-ACTGGATCTTAAGGCTCAGGAT  *r*-GACTTAATGTCACTGTTCAGCG | 572 | 58 | (5) |
| ETEC | *eatA* | *f*-CAGGAGTGGGAACATTAAGTCA  *r*-CGTACGCCTTTGATTTCAGGAT | 743 | 58 | (5) |
| EHEC | *espP* | *f*-GTCCATGCAGGGACATGCCA  *r*-TCACATCAGCACCGTTCTCTAT | 547 | 58 | (5) |
| EHEC | *pks* | *f*-GCGCATCCTCAAGAGTAAATA  GCGCTCTATGCTCATCAACC | 280 | 58 | (7) |
| EHEC | *cnf* | *f*-GGGGGAAGTACAGAAGAATTA  *CNF1as*-TTGCCGTCCACTCTCACCAGT | 1112 | 55 | (8) |
| EPEC | *cif* | *f*-AACAGATGGCAACAGACTGG  *r*-AGTCAATGCTTTATGCGTCAT | 383 | 55 | (8) |
| *E. coli C0309* | *ybtS* | *f*-GACGGAAACAGCACGGTAAA  *r*-GAGCATAATAAGGCGAAAGA | 242 | 60 | (9) |
| *E. coli C0428* | *entB* | *f*-GTCAACTGGGCCTTTGAGCCGTC  *r*-TATGGGCGTAAACGCCGGTGAT | 400 | 60 | (9) |
| EAEC | *chuA* | *f*-GACGAACCAACGGTCAGGAT  *r*-TGCCGCCAGTACCAAAGACA | 272 | 55 | (10) |
| EPEC | *yjaA* | *f*-TGAAGTGTCAGGAGACGCTG  *f*-ATGGAGAATGCGTTCCTCAAC | 211 | 55 | (10) |
| DAEC | TspE4.C2 | *f*-GAGTAATGTCGGGGCATTCA  *f*-CGCGCCAACAAAGTATTACG | 152 | 55 | (10) |

1. Vidal R, Vidal M, Lagos R, Levine M, Prado V. Multiplex PCR for diagnosis of enteric infections associated with diarrheagenic Escherichia coli. J Clin Microbiol. 2004;42(4):1787-9.

2. Canizalez-Roman A, Gonzalez-Nunez E, Vidal JE, Flores-Villasenor H, Leon-Sicairos N. Prevalence and antibiotic resistance profiles of diarrheagenic Escherichia coli strains isolated from food items in northwestern Mexico. Int J Food Microbiol. 2013;164(1):36-45.

3. Vidal M, Kruger E, Duran C, Lagos R, Levine M, Prado V, et al. Single multiplex PCR assay to identify simultaneously the six categories of diarrheagenic Escherichia coli associated with enteric infections. J Clin Microbiol. 2005;43(10):5362-5.

4. Angulo-Zamudio UA, Gutierrez-Jimenez J, Monroy-Higuera L, Flores-Villasenor H, Leon-Sicairos N, Velazquez-Roman J, et al. Non-diarrheagenic and diarrheagenic E. coli carrying supplementary virulence genes (SVG) are associated with diarrhea in children from Mexico. Microb Pathog. 2021;157:104994.

5. Restieri C, Garriss G, Locas MC, Dozois CM. Autotransporter-encoding sequences are phylogenetically distributed among Escherichia coli clinical isolates and reference strains. Appl Environ Microbiol. 2007;73(5):1553-62.

6. Boisen N, Ruiz-Perez F, Scheutz F, Krogfelt KA, Nataro JP. Short report: high prevalence of serine protease autotransporter cytotoxins among strains of enteroaggregative Escherichia coli. Am J Trop Med Hyg. 2009;80(2):294-301.

7. Arthur JC, Perez-Chanona E, Muhlbauer M, Tomkovich S, Uronis JM, Fan TJ, et al. Intestinal inflammation targets cancer-inducing activity of the microbiota. Science. 2012;338(6103):120-3.

8. Dubois D, Delmas J, Cady A, Robin F, Sivignon A, Oswald E, et al. Cyclomodulins in urosepsis strains of Escherichia coli. J Clin Microbiol. 2010;48(6):2122-9.

9. Compain F, Babosan A, Brisse S, Genel N, Audo J, Ailloud F, et al. Multiplex PCR for detection of seven virulence factors and K1/K2 capsular serotypes of Klebsiella pneumoniae. J Clin Microbiol. 2014;52(12):4377-80.

10. Clermont O, Bonacorsi S, Bingen E. Rapid and simple determination of the Escherichia coli phylogenetic group. Appl Environ Microbiol. 2000;66(10):4555-8.
