# Supplementary material for: Virulence genes, antimicrobial resistance profile, phylotyping and pathotyping of diarrheagenic Escherichia coli isolated from children in Southwest Mexico
Source: PLoS One. 2024 Mar 12;19(3):e0300304. doi: 10.1371/journal.pone.0300304 (PMC10931464; doi:10.1371/journal.pone.0300304)
Supplement: S3 Table — (DOCX) [file pone.0300304.s003.docx]

| **Supplementary Table 3. PCR profile of isolate by isolate.** | | | | | | | |
| --- | --- | --- | --- | --- | --- | --- | --- |
|  | **Pathotypes *E. coli*** | | | | | | |
|  |  |  |  |  |  |  |  |
| **ID** | **EAEC** | **aEPEC** | **tEPEC** | **ETEC** | **DAEC** | **EIEC** | **EHEC** |
|  | *aafll* | *eae* | *eae/bfp* | *lt/st* | *daaE* | *ipaH/virF* | *eae/stx1/stx2* |
| 1165 | - | - | - | + | - | - | - |
| 1245 | - | - | - | - | + | - | - |
| 2415 | - | + | - | - | - | - | - |
| 3543 | + | - | - | - | - | - | - |
| 3686 | + | + | - | - | - | - | - |
| 4272 | - | + | - | - | - | - | - |
| 4621 | + | - | - | - | - | - | - |
| 4815 | - | - | - | - | + | - | - |
| 4816 | + | - | - | - | - | - | - |
| 4817 | - | + | - | - | - | - | - |
| 5001 | + | + | - | - | - | - | - |
| 5022 | - | + | - | + | - | - | - |
| 5023 | - | + | - | - | - | - | - |
| 5024 | - | + | - | - | - | - | - |
| 5026 | - | + | - | - | - | - | - |
| 5027 | - | + | - | - | - | - | - |
| 5608 | + | - | - | - | - | - | - |
| 5614 | - | + | - | - | - | - | - |
| 5869 | - | - | - | - | + | - | - |
| 5973 | - | + | - | - | - | - | - |
| 6151 | - | + | - | - | - | + | - |
| 6226 | - | + | - | - | - | - | - |
| 6350 | - | + | - | - | - | - | - |
| 6367 | - | - | - | + | - | - | - |
| 6553 | - | + | - | - | - | - | - |
| 6910 | - | - | + | - | - | - | - |
| 6912 | - | + | - | + | - | - | - |
| 7049 | - | + | - | - | + | - | - |
| 7105 | - | + | - | - | - | - | - |
| 7142 | - | + | - | - | - | + | - |
| 7143 | - | + | - | - | - | - | - |
| 7752 | - | - | + | - | - | - | - |
| 7765 | - | - | + | - | - | + | - |
| 7766 | - | + | + | - | - | - | - |
| 8635 | - | + | - | - | - | - | - |
| 8736 | - | + | - | - | - | + | - |
| 9642 | - | - | - | - | + | - | - |
| 9753 | + | - | - | - | - | - | - |
| 9802 | + | - | - | - | - | - | - |
| 9997 | + | - | - | - | - | - | - |
| 10275 | - | + | - | - | - | - | - |
| 10530 | - | - | - | - | + | - | - |
| 2689 | - | + | - | + | - | - | - |
| 3631 | - | + | - | - | - | - | - |
| 3749 | + | + | - | - | - | - | - |
| 3996 | + | - | - | - | - | - | - |
| 4882 | - | + | - | - | - | - | - |
| 4883 | - | + | - | - | - | - | - |
| 4961 | + | - | - | - | - | - | - |
| 5796 | - | - | + | - | - | - | - |
| 5798 | - | + | - | - | + | - | - |
| 6742 | - | + | - | - | - | - | - |
| 6801 | - | + | - | - | - | - | - |
| 7302 | - | - | - | - | - | + | - |
| 7304 | - | - | - | + | - | - | - |
| 7452 | - | + | - | - | - | - | - |
| 7453 | - | + | - | - | - | - | - |
| 7458 | - | + | - | - | - | - | - |
| 445 | + | - | - | - | - | - | - |
| 961 | - | + | - | - | - | - | - |
| 3533 | - | + | - | - | - | - | - |
| 3534 | - | + | - | - | - | - | - |
| 3924 | - | + | - | - | - | - | - |
| 4032 | - | - | - | - | + | - | - |
| 4727 | - | + | - | - | - | - | - |
| 4729 | + | - | - | - | - | - | - |
| 5242 | - | + | - | - | - | - | - |
| 5247 | - | + | - | - | - | - | - |
| 6237 | - | + | - | - | - | - | - |
| 6240 | - | + | - | - | - | - | - |
| 6944 | - | + | - | - | - | - | - |
| 7771 | - | + | - | - | - | - | - |
| 7772 | - | + | - | - | - | - | - |
| 8055 | - | + | - | - | + | - | - |
| 8469 | - | + | - | - | + | - | - |
| 8470 | - | + | - | - | - | - | - |
| 8471 | - | + | - | - | - | - | - |
| 8479 | + | - | - | - | - | - | - |
| 10271 | - | - | - | + | - | - | - |
| 328 | - | + | - | - | - | - | - |
| 394 | + | - | - | - | - | - | - |
| 858 | - | + | - | - | - | - | - |
| 946 | - | - | - | - | + | - | - |
| 1985 | - | + | - | - | - | - | - |
| 2329 | - | + | + | - | - | + | - |
| 2772 | - | + | - | - | - | - | - |
| 2908 | - | + | - | + | - | - | - |
| 3343 | - | + | - | - | - | - | - |
| 3344 | - | + | - | - | - | - | - |
| 3346 | - | + | - | - | - | - | - |
| 3348 | + | + | - | - | - | - | - |
| 3421 | - | + | - | - | - | - | - |
| 3464 | - | + | - | - | - | - | - |
| 3501 | - | + | + | - | - | - | - |
| 3503 | - | + | - | - | - | - | - |
| 3504 | + | + | - | - | - | - | - |
| 3759 | - | + | - | - | + | - | - |
| 3953 | + | + | - | - | - | - | - |
| 4144 | + | - | - | - | - | - | - |
| 5050 | - | + | - | - | - | - | - |
| 6604 | + | + | - | - | - | - | - |
| 6605 | - | + | - | - | - | - | - |
| 6739 | - | + | - | - | - | - | - |
| 6744 | - | + | - | - | - | - | - |
| 8227 | - | - | + | - | - | - | - |
| 9924 | - | + | - | - | - | - | - |
| 10476 | - | + | - | - | - | + | - |
| 1575 | - | - | - | + | - | - | - |
| 2232 | - | - | - | + | - | - | - |
| 3779 | - | + | - | - | - | - | - |
| 3785 | - | + | - | - | - | - | - |
| 4283 | + | + | - | - | - | - | - |
| 4290 | + | - | - | - | - | - | - |
| 5891 | - | + | - | - | - | - | - |
| 6215 | - | + | - | - | - | - | - |
| 6216 | + | + | - | - | - | - | - |
| 6504 | - | - | + | - | - | - | - |
| 6511 | - | + | - | - | - | - | - |
| 6512 | - | + | - | - | - | - | - |
| 6513 | + | + | - | + | - | - | - |
| 6515 | + | + | - | - | - | - | - |
| 6516 | - | + | - | - | - | - | - |
| 6519 | - | + | - | - | + | - | - |
| 6520 | - | + | - | - | - | - | - |
| 6528 | - | + | - | - | - | - | - |
| 6529 | - | + | - | - | - | - | - |
| 6531 | + | + | - | - | - | - | - |
| 6532 | - | + | - | + | - | - | - |
| 6535 | - | + | - | - | - | - | - |
| 6540 | - | + | - | - | - | - | - |
| 6542 | - | + | - | - | - | - | - |
| 6543 | - | + | - | - | - | - | - |
| 6545 | - | + | - | - | - | - | - |
| 7206 | - | + | - | - | - | - | - |
| 7207 | - | + | - | - | - | - | - |
| 7216 | + | - | - | - | + | - | - |
| 7653 | - | + | - | - | - | - | - |
| 7656 | - | - | + | - | - | - | - |
| 7658 | - | - | + | - | - | - | - |
| 7684 | - | + | + | - | - | - | - |
| 7691 | - | + | - | - | - | - | - |
| 7701 | - | - | + | - | - | - | - |
| 2478 | - | - | - | - | - | + | - |
| 5372 | - | + | - | - | - | - | - |
| 5605 | - | + | - | - | - | - | - |
| 5674 | - | + | - | - | - | - | - |
| 6165 | - | - | - | - | - | - | + |
