# Supplementary material for: Virulence genes, antimicrobial resistance profile, phylotyping and pathotyping of diarrheagenic Escherichia coli isolated from children in Southwest Mexico
Source: PLoS One. 2024 Mar 12;19(3):e0300304. doi: 10.1371/journal.pone.0300304 (PMC10931464; doi:10.1371/journal.pone.0300304)
Supplement: Attachment — Submitted filename: PONE-D-23-33103_reviewer highlighted.pdf [file pone.0300304.s004.pdf]

PLOS ONE

Diarrheagenic Escherichia coli are isolated primarily from children under five years of age with diarrhea in low-income areas of Mexico: Supplementary virulence genes (SVG) and antimicrobial resistance.

--Manuscript Draft--

Manuscript Number:	PONE-D-23-33103
Article Type:	Research Article
Full Title:	Diarrheagenic Escherichia coli are isolated primarily from children under five years of age with diarrhea in low-income areas of Mexico: Supplementary virulence genes (SVG) and antimicrobial resistance.
Short Title:	Diarrheagenic E. coli are isolated primarily from children with diarrhea in low-income areas of Mexico
Corresponding Author:	Gabriela Tapia-Pastrana, PhD<br>HRAEO: Hospital Regional de Alta Especialidad de Oaxaca<br>Oaxaca, Oaxaca MEXICO
Keywords:	E. coli; DEC; diarrhea; Supplemental virulence genes; Resistance
Abstract:	Diarrheagenic E. coli (DEC) strains are one of the most important etiological factors causing diarrhea in children worldwide, especially in developing countries. DEC strains contain characteristic virulence factors; however, other supplemental virulence genes (SVG) may contribute to the development of diarrhea in children. Therefore, this study aimed to determine the prevalence of DEC in children with diarrhea in southwestern Mexico and to associate childhood symptoms, SVG, and pathotypes with diarrhea-causing DEC strains. DEC strains were isolated from 230 children with diarrhea aged 0-60 months from the state of Oaxaca, southwestern Mexico; clinical data were collected, and PCR was used to identify SVG and pathotypes. Antibiotic resistance profiling was performed on DEC strains. 63% of samples were DEC positive, single or combined infections (two or three strains) of aEPEC, EAEC, DAEC, tEPEC, ETEC, EIEC, or EHEC were found, with aEPEC being the most common. Children aged ≤ 12 and 49-60 months and symptoms (e.g., fever and blood) were associated with DEC strains. SVG related to colonization (nleB), cytotoxicity (sat and espC), and proteolysis (pic) were associated with DEC strains, whereas espC, sat, and pic were associated with E. coli pathotypes. Over 79% of the DEC strains were resistant to antibiotics, and 40% were MDR and XDR, respectively. In conclusion aEPEC was the most prevalent pathotype in children with diarrhea in this region. SVG related to colonization, cytotoxicity, and proteolysis were associated with diarrhea-producing DEC strains, which may play an essential role in the development of diarrhea in children in southwestern Mexico.
Order of Authors:	Gabriela Tapia-Pastrana, PhD
	Metztli Rojas-Bautista, Msc
	Pilar Hernández-Pérez
	Olegario Santiago-Martínez
	Lucía C. Gómez-Rodríguez
	Víctor M. Terrazas-Luna
	Jacobo Montes-Yedra
	Alfonso A. Bautista-Avendaño
	Eduardo S. García-López
	Nidia Leon-Sicairos, PhD
	Uriel A. Angulo-Zamudio, PhD
	Adrian Canizalez-Roman, PhD

Powered by Editorial Manager® and ProduXion Manager® from Aries Systems Corporation

Additional Information:	
**Question**	**Response**
**Financial Disclosure**<br><br>Enter a financial disclosure statement that describes the sources of funding for the work included in this submission. Review the submission guidelines for detailed requirements. View published research articles from *PLOS ONE* for specific examples.<br><br>This statement is required for submission and **will appear in the published article** if the submission is accepted. Please make sure it is accurate.<br><br>**Funded studies**<br>Enter a statement with the following details:<br>• Initials of the authors who received each award<br>• Grant numbers awarded to each author<br>• The full name of each funder<br>• URL of each funder website<br>• Did the sponsors or funders play any role in the study design, data collection and analysis, decision to publish, or preparation of the manuscript?<br><br>Did you receive funding for this work?	Yes
Please add funding details.<br>as follow-up to "**Financial Disclosure**<br><br>Enter a financial disclosure statement that describes the sources of funding for the work included in this submission. Review the submission guidelines for detailed requirements. View published research articles from *PLOS ONE* for specific examples.<br><br>This statement is required for submission and **will appear in the published article** if the submission is accepted. Please make sure it is accurate.	This work was supported by grants #25501 and #25101 from the Secretaría de Salud through the Hospital Regional de Alta Especialidad de Oaxaca to G.T.P

**Funded studies**<br>Enter a statement with the following details:<br>• Initials of the authors who received each award<br>• Grant numbers awarded to each author<br>• The full name of each funder<br>• URL of each funder website<br>• Did the sponsors or funders play any role in the study design, data collection and analysis, decision to publish, or preparation of the manuscript?<br><br>Did you receive funding for this work?"	
Please select the country of your main research funder (please select carefully as in some cases this is used in fee calculation).<br>as follow-up to "**Financial Disclosure**<br><br>Enter a financial disclosure statement that describes the sources of funding for the work included in this submission. Review the submission guidelines for detailed requirements. View published research articles from *PLOS ONE* for specific examples.<br><br>This statement is required for submission and **will appear in the published article** if the submission is accepted. Please make sure it is accurate.<br><br>**Funded studies**<br>Enter a statement with the following details:<br>• Initials of the authors who received each award<br>• Grant numbers awarded to each author<br>• The full name of each funder<br>• URL of each funder website<br>• Did the sponsors or funders play any role in the study design, data collection and analysis, decision to publish, or preparation of the manuscript?<br><br>Did you receive funding for this work?"	MEXICO - MX
**Competing Interests**	The authors have declared that no competing interests exist.

Use the instructions below to enter a competing interest statement for this submission. On behalf of all authors, disclose any competing interests that could be perceived to bias this work—acknowledging all financial support and any other relevant financial or non-financial competing interests.<br><br>This statement is required for submission and **will appear in the published article** if the submission is accepted. Please make sure it is accurate and that any funding sources listed in your Funding Information later in the submission form are also declared in your Financial Disclosure statement.<br><br>View published research articles from *PLOS ONE* for specific examples.<br><br>**NO authors have competing interests**<br><br>Enter: *The authors have declared that no competing interests exist.*<br><br>**Authors with competing interests**<br><br>Enter competing interest details beginning with this statement:<br><br>*I have read the journal's policy and the authors of this manuscript have the following competing interests: [insert competing interests here]*<br><br>* typeset	
**Ethics Statement**<br><br>Enter an ethics statement for this submission. This statement is required if the study involved:<br><br>• Human participants<br>• Human specimens or tissue<br>• Vertebrate animals or cephalopods<br>• Vertebrate embryos or tissues	Ethics approval:<br>The study was approved by the Ethics Committee of the HRAEO project No. HRAEO-CIC-CEI 017/14.<br>Consent to participate and for publication: All participants were informed about the project and gave verbal and written consent (the parents) to participate.

Powered by Editorial Manager® and ProduXion Manager® from Aries Systems Corporation

• Field research<br><br>Write "N/A" if the submission does not require an ethics statement.<br><br>General guidance is provided below. Consult the submission guidelines for detailed instructions. **Make sure that all information entered here is included in the Methods section of the manuscript.**	

**Format for specific study types**<br><br>**Human Subject Research (involving human participants and/or tissue)**<br>• Give the name of the institutional review board or ethics committee that approved the study<br>• Include the approval number and/or a statement indicating approval of this research<br>• Indicate the form of consent obtained (written/oral) or the reason that consent was not obtained (e.g. the data were analyzed anonymously)<br><br>**Animal Research (involving vertebrate animals, embryos or tissues)**<br>• Provide the name of the Institutional Animal Care and Use Committee (IACUC) or other relevant ethics board that reviewed the study protocol, and indicate whether they approved this research or granted a formal waiver of ethical approval<br>• Include an approval number if one was obtained<br>• If the study involved *non-human primates*, add *additional details* about animal welfare and steps taken to ameliorate suffering<br>• If anesthesia, euthanasia, or any kind of animal sacrifice is part of the study, include briefly which substances and/or methods were applied<br><br>**Field Research**<br><br>Include the following details if this study involves the collection of plant, animal, or other materials from a natural setting:<br>• Field permit number<br>• Name of the institution or relevant body that granted permission	
**Data Availability**<br><br>Authors are required to make all data underlying the findings described fully available, without restriction, and from the time of publication. PLOS allows rare exceptions to address legal and ethical concerns. See the PLOS Data Policy and FAQ for detailed information.	No - some restrictions will apply

A Data Availability Statement describing where the data can be found is required at submission. Your answers to this question constitute the Data Availability Statement and **will be published in the article**, if accepted.

Important: Stating 'data available on request from the author' is not sufficient. If your data are only available upon request, select 'No' for the first question and explain your exceptional situation in the text box.

Do the authors confirm that all data underlying the findings described in their manuscript are fully available without restriction?

Describe where the data may be found in full sentences. If you are copying our sample text, replace any instances of XXX with the appropriate details.

- If the data are **held or will be held in a public repository**, include URLs, accession numbers or DOIs. If this information will only be available after acceptance, indicate this by ticking the box below. For example: *All XXX files are available from the XXX database (accession number(s) XXX, XXX.).*
- If the data are all contained **within the manuscript and/or Supporting Information files**, enter the following: *All relevant data are within the manuscript and its Supporting Information files.*
- If neither of these applies but you are able to provide **details of access elsewhere**, with or without limitations, please do so. For example:

  Data cannot be shared publicly because of [XXX]. Data are available from the XXX Institutional Data Access / Ethics Committee (contact via XXX) for researchers who meet the criteria for access to confidential data.

  The data underlying the results presented in the study are available from (include the name of the third party

All relevant data are within the manuscript and its Supporting Information files.

Powered by Editorial Manager® and ProduXion Manager® from Aries Systems Corporation

and contact information or URL).<br>• This text is appropriate if the data are owned by a third party and authors do not have permission to share the data.<br><br>* typeset	
Additional data availability information:	

Powered by Editorial Manager® and ProduXion Manager® from Aries Systems Corporation

Manuscript

Click here to access/download;Manuscript;DEC and SVG and Oaxaca-Oct-10-plos one.docx

Diarrheagenic *Escherichia coli* are isolated primarily from children under five years of age with diarrhea in low-income areas of Mexico: Supplementary virulence genes (SVG) and antimicrobial resistance.

Gabriela Tapia-Pastrana[1]*, Metztli Rojas-Bautista[2], Pilar Hernández-Pérez[2], Olegario Santiago-Martínez[2], Lucía C. Gómez-Rodríguez[2], Víctor M. Terrazas-Luna[1], Jacobo Montes-Yedra[3], Alfonso A. Bautista-Avendaño[3], Eduardo S. García-López[4], Nidia Leon-Sicairos[5,6], Uriel A. Angulo-Zamudio[5], Adrian Canizalez-Roman[5,7].

[1]*Laboratorio de Investigación Biomédica, Hospital Regional de Alta Especialidad de Oaxaca, San Bartolo Coyotepec, Oaxaca 71256, Mexico;* [2]*Facultad de Ciencias Químicas de la Universidad Autónoma Benito Juárez de Oaxaca, 68110 Oaxaca de Juarez, Oaxaca Mexico;* [3]*Departamento de Ciencias Básicas del Instituto Tecnológico del Valle de Oaxaca, 71233 Santa Cruz Xoxocotlán, Oaxaca Mexico;* [4]*Laboratorio Estatal de Salud Publica Oaxaca, 71257 Reyes Mantecón, Oaxaca Mexico;* [5]*School of Medicine, Autonomous University of Sinaloa, 80246 Culiacan Sinaloa, Mexico;* [6]*Pediatric Hospital of Sinaloa, 80200 Culiacan Sinaloa, Mexico;* [7]*The Women's Hospital, Secretariat of Health, 80020 Culiacan Sinaloa, Mexico.*

***Corresponding author;** *Laboratorio de Investigación Biomédica, Hospital Regional de Alta Especialidad de Oaxaca, San Bartolo Coyotepec, Oaxaca 71256, Mexico.* Phone: (52), Fax: (52). E-mail: gtapia@hraeoaxaca.gob.mx

Conflicts of interest: None of the authors have any proprietary interests or conflicts of interest related to this submission.

This submission has not been published anywhere previously, and it is not simultaneously being considered for any other publication.

Running title: *Diarrheagenic E. coli are associated with childhood diarrhea in low-income areas of Mexico.*

Abstract

Diarrheagenic *E. coli* (DEC) strains are one of the most important etiological factors causing diarrhea in children worldwide, especially in developing countries. DEC strains contain characteristic virulence factors; however, other supplemental virulence genes (SVG) may contribute to the development of diarrhea in children. Therefore, this study aimed to determine the prevalence of DEC in children with diarrhea in southwestern Mexico and to associate childhood symptoms, SVG, and pathotypes with diarrhea-causing DEC strains. DEC strains were isolated from 230 children with diarrhea aged 0-60 months from the state of Oaxaca, southwestern Mexico; clinical data were collected, and PCR was used to identify SVG and pathotypes. Antibiotic resistance profiling was performed on DEC strains. 63% of samples were DEC positive, single or combined infections (two or three strains) of aEPEC, EAEC, DAEC, tEPEC, ETEC, EIEC, or EHEC were found, with aEPEC being the most common. Children aged ≤ 12 and 49-60 months and symptoms (e.g., fever and blood) were associated with DEC strains. SVG related to colonization (*nleB*), cytotoxicity (*sat* and *espC*), and proteolysis (*pic*) were associated with DEC strains, whereas *espC*, sat, and pic were associated with *E. coli* pathotypes. Over 79% of the DEC strains were resistant to antibiotics, and 40% were MDR and XDR, respectively. In conclusion aEPEC was the most prevalent pathotype in children with diarrhea in this region. SVG related to colonization, cytotoxicity, and proteolysis were associated with diarrhea-producing DEC strains, which may play an essential role in the development of diarrhea in children in southwestern Mexico.

Keywords: *E. coli*; DEC; Diarrhea; Supplemental virulence genes; Resistance**.**

Introduction

Diarrhea is a serious public health problem and a significant cause of child morbidity and mortality. Developing countries are most affected by this disease, including those in Africa, Asia, and Latin America, with most cases associated with poor living conditions, such as poor environmental hygiene and sanitation, poor water conditions, and poor cooking practices, among others [1]. Several etiological agents cause diarrhea; however, diarrheagenic *E. coli* (DEC) is one of the most common.

DEC are classified according to their production of virulence factors, some related to the adhesion process, cytotoxic capacity, or whether they contain a capsule [2]. The presence of specific virulence factor genes classifies DEC into six different pathotypes. Enteropathogenic *E. coli* (EPEC) strains are those that contain the locus of enterocyte effacement (LEE) pathogenicity island, which encodes an outer membrane adhesion protein (eaeA+), and a plasmid called EPEC adhesion factor (EAF). Depending on the absence of EAF (LEE+ and A/E+ phenotype), EPEC can be classified as atypical [3]. Enterotoxigenic *E. coli* (ETEC) strains are those that are positive for enterotoxins such as thermolabile and thermostable toxins (LT and ST) [2, 4]. Enteroinvasive *E. coli* (EIEC) strains are *virF* transcription activators and type three secretion system (T3SS) positive [5]. Enteroaggregative *E. coli* (EAEC) strains have an aggregative adherence (AA) characteristic; in addition, this pathotype is positive for the AggR regulon [6, 7]. Diffusely adherent *E. coli* (DAEC) strains have a diffuse pattern of adherence in cells; in addition, they are positive for some adhesins: afimbrial (Afa) or fimbrial (Dr) [8]. Vero toxin-producing/Shiga toxin-producing *E. coli* (VTEC/STEC) are those diarrhea-causing strains that encode for Shiga toxin genes (*stx1* and/or *stx2*) [9]. Enterohemorrhagic *E. coli* (EHEC) strains this is a subgroup of STEC; in addition to the *stx* genes, these strains also carry the *eae* gene, which encodes an intimin; this protein plays a central role in EHEC lesion fixation and eradication [4, 8].

In addition to the virulence factors that characterize each DEC pathotype, some supplementary virulence genes (SVG) have recently been associated with the ability of DEC to cause

diarrhea and with the higher virulence phenotype of these strains [10]. SVG are defined as genes encoding proteins involved in (i) colonization (*ehaC*, *ehaD*, *cah*, and *nleB*, which are associated with adhesins or biofilm formation [11, 12]), (ii) cytotoxicity (*sat*, *espC*, *pet*, *hylA*, and *stx1*, these toxins are associated with damage to the cytoskeleton, tight junctions or ribosome of epithelial cells [13, 14], (iii) proteolysis (*pic*, *eatA*, and *espP*, which encode proteins with the ability to degrade mucin, spectrin, pepsin or plasma proteins [15, 16]), (iv) cyclomodulins (*cnf*, *pks*, and *cif*, which encode proteins with the ability to cause DNA damage, modulate the cell cycle, and induce epithelial cell tumors [17]), and v) iron acquisition (*ybtS*, and *entB*, these genes have activities related to siderophores and iron metabolism [18]).

Another essential feature of DEC is the high antibiotic resistance of these bacteria. DEC isolated from children with diarrhea has increased antibiotic resistance over the years; for example, in the early 2000s, European countries reported zero resistance to trimethoprim, but in 2016, this resistance increased to 28.8%. Antibiotic resistance in *E. coli* also varies across nations; in Organization for Economic Cooperation and Development (OECD) countries, *E. coli* resistance ranged from 0.3% to 37.7%, while in non-OECD countries, *E. coli* antibiotic resistance ranged from 5% to 80% [19, 20]. Antibiotic resistance is a significant public health problem that is increasing yearly, so more strategies must be developed to address this problem.

DEC strains are associated with cases of diarrhea, but the population most affected by these bacteria are children. Cases of DEC diarrhea in children have been reported worldwide in the United States of America [21], Mexico [22], South America [23], Europe [24], and Asia [25]. However, studies have shown that DEC may also require SVG to develop cases of diarrhea; a study conducted in the state of Yucatan showed that DEC with SVG (*aap*, *aatA*, *astA*, *pet*, and *cdt*) resulted in more cases of moderate or severe diarrhea in children [26]. In addition, our work team showed that DEC were associated with diarrhea when the strains carried an SVG (*ehaC*, *kps*, *nleB*, and/or *espC*) in children in northwestern Mexico [27].

On the other hand, Oaxaca is one of the three poorest states in Mexico, and the cause of DEC diarrhea, DEC-associated signs, and symptoms, and DEC-associated SVG leading to diarrhea may differ from the rest of the Mexican states. Therefore, this comprehensive study evaluated the prevalence of DEC in Oaxacan children aged 0 to 60 months, symptoms, SVG, pathotypes, and their association with diarrhea-causing DEC strains. Finally, the antibiotic resistance profiles of the DEC strains were studied.

Materials and Methods.

Sample collection.

From January 1, 2016 to January 31, 2017, a total of 230 stool samples were collected from children aged 0-5 years of both sexes with diarrhea from six health jurisdictions in the state of Oaxaca located in southwestern Mexico: Valles Centrales (J1), Istmo (J2), Tuxtepec (J3), Costa (J4), Mixteca (J5), and Sierra Sur (J6) (Fig. 1). The state of Oaxaca is one of the most impoverished in Mexico, and the population of these six jurisdictions represents ~99% of the state's inhabitants. Patients with acute diarrhea came to primary care units where they were evaluated by licensed physicians who confirmed that patients had the passage of three or more loose or liquid stools per day or more frequent passage than is usual. In addition, other symptoms were recorded from electronic medical records: fever, vomiting, dehydration, abdominal pain, and watery diarrhea with mucus and/or blood. Rectal swabs were collected by standard procedures for routine diagnostics at the primary care units and transferred to the Cary-Blair transport medium. Swabs were stored at 4°C at the communal health station until transportation in cold boxes to the Public Health Laboratory of the State of Oaxaca and the Biomedical Research Laboratory of the Hospital Regional de Alta Especialidad de Oaxaca (HRAEO), usually within 2 hours. For the sample collection, the children who participated in the project, the parents signed an informed consent form explaining the sample collection procedures, and the study was approved by the Ethics Committee of the HRAEO, which approved this project No. HRAEO-CIC-CEI 017/14.

Isolation and identification of *E. coli*.

Samples were streaked on MacConkey agar for 24 hours at 37°C, and colonies with *E. coli*-related characteristics were subjected to the following conventional biochemical tests: catalase test

(+), oxidase test (-), glucose fermentation with gas production. In addition, the biochemical test was performed using the API20E kit (Biomerieux, Marcy-l'Etoile, France) according to the manufacturer's instructions. In addition, a PCR assay was performed to identify the 16SrRNA gene of *E. coli* for molecular identification. The sequence of the primers and the size of the PCR products are shown in Supplementary Table 1. Finally, *E. coli* isolates were subcultured on Luria-Bertani (LB) agar and stored in LB broth plus 20% glycerol at -70 °C for further experiments.

Bacterial Strains.

The reference strains used in this study were from our laboratory collection. EPEC E2348/69 (*eae*+ and *bfp*+), EHEC O157:H7 EDL933 (*eae*+, *stx1*+ and *stx2*+), EIEC EI-34 (*ipaH*+ and *virF*+), EAEC O42 (*aafII*+), ETEC H10407 (*lt*+) and DAEC F1845 (*daaE*+) were used to identify DEC. For the identification of SVG, cyclomodulins and siderophores, strains used in previous studies of our group were used as controls [27, 28]. In addition, the most representative amplicons were sequenced for control purposes.

Preparation of template DNA.

The boiling method was used to extract genomic DNA. Three bacterial colonies from each isolate were vortexed in a 1.5 mL tube containing nuclease-free water. After boiling for 5 minutes, the mixture was immediately placed in a freezer for 2 minutes. The suspension was then centrifuged (12,000 × g) for 5 min. From the supernatant (containing the extracted DNA), 150 µL of the sample was transferred to a new 0.6 mL tube. The DNA was stored at -20°C for further experiments.

Molecular Identification of DEC Strains.

DEC strains among *E. coli* isolates were identified using a multiplex and singleplex PCR reaction scheme based on previous work [29]: Multiplex PCR reaction #1 contained primers to

amplify the intimin gene (*eae*) and the structural subunit of the bundle-forming pilus gene (*bfp*), and detected both typical (*eae*+ and *bfp*+) and atypical (*eae*+ and *bfp*-) EPEC strains. Multiplex PCR reaction #2 detected EHEC strains and included primers to amplify the *eae* gene and the Shiga toxin genes *stx1* and *stx2*. EIEC strains were detected with reaction #3, which included primers to amplify the invasion genes *virF* and *ipaH*. EAEC strains were detected by amplification of the *aafll* gene using singleplex PCR reaction number 4. ETEC strains were detected using reaction number 5, which contained primers to amplify the *lt* gene. Finally, singleplex PCR reaction number 6 had primers amplifying the *daaE* gene to detect DAEC strains. The sequences of the primers and the sizes of the PCR products with these targets are shown in Supplementary Table 1. For all PCR reactions, we mixed MyTaq (BIOLINE, Tennessee, USA), 0.5 µM direct and reverse primers, and 2 µL genomic DNA and molecular biology grade water, and the reactions were performed on a Mastercycler flexlid thermal cycler (Eppendorf, Hamburg, Germany). PCR products were visualized with ethidium bromide, and the results were analyzed on a PhotoDoc-It imaging system with a Benchtop 2 UV transilluminator (UVP). All DEC-negative specimens were excluded from the study.

Molecular identification of the phylogenetic groups.

Phylogenetic groups A, B1, B2, and D were identified as described by Clermont et al. We performed singleplex PCR reactions to identify the *chuA*, *yjaA*, and *tspE4.C2* genes (Clermont, Bonacorsi, and Bingen, 2000). Primers sequences and PCR product sizes for these targets are listed in Supplementary Table 2.

Detection of SVG, Cyclomodulins, and Siderophores by PCR.

The SVG targets were divided into three groups based on the putative role of the proteins encoded by each gene in virulence. The SVG related to colonization were *ehaC*, *ehaD*, *cah*, and *nleB*. Those related to cytotoxicity: *pet*, *espC*, *sat*, *hlyA* and *stx1*. Those related to proteolysis: *pic*, *eatA*, and *espP*. Cyclomodulins were also identified: Pks, CNF, and CIF (*clbB*, *cnf*, and *cif* were used as

target genes). Finally, the siderophore-associated genes *ybtS* and *entB* were identified. Primers sequences and PCR product sizes are listed in Supplementary Table 1.

Cytotoxicity assay.

Cytotoxicity was evaluated in HEp-2 cells (laryngeal carcinoma cells) using isolates of the different DEC pathotypes with randomly selected SVG. HEp-2 cells were cultured in DMEM medium (Corning) supplemented with 10% fetal bovine serum and antibiotics [penicillin 100 U/mL, streptomycin 100 µg/mL] (Gibco). Cells were incubated in a 5% CO2 atmosphere at 37°C until 90% confluence was reached. HEp-2 cells were then detached with 0.25% trypsin, EDTA, and 1X sodium bicarbonate (Corning), resuspended in supplemented DMEM, and seeded in a 96-well box (Costar 3590, CORNING) at a density of 30,000 cells/mL and grown to monolayer confluence using the incubation conditions described above. Infection was performed at a multiplicity of infection (MOI) of 10 for 12 h in a 5% CO2 atmosphere at 37°C. *E. coli* HB101 was used as a negative control for cytotoxicity, while buffer lysis was used as a positive control. Cytotoxicity was quantified by LDH release using the CytoTox 96® kit (PROMEGA) according to the manufacturer's instructions.

Antimicrobial Susceptibility Testing.

Antimicrobial susceptibility testing of pathogenic isolates was performed using the Kirby-Bauer disk diffusion method according to the guidelines developed by the Clinical Laboratory Standard Institute, following the methodology of previous work [27]. Suspensions of *E. coli* isolates were prepared in LB at 0.5 turbidity using the McFarland standard. These cultures were then seeded on Mueller-Hinton agar plates, and antibiotic discs (BD BBL, Franklin Lakes, NJ, USA) were aseptically applied to the inoculated agar. The antibiotics tested were ampicillin, amoxicillin/clavulanic acid, ampicillin/sulbactam, cefazolin, cefotaxime, ceftriaxone, ceftazidime, aztreonam, imipenem, kanamycin, streptomycin, tetracycline, ciprofloxacin, nalidixic acid, trimethoprim, and chloramphenicol. Plates were incubated at 37 ◦C for 18 h. The diameters (in millimeters) of the clear

growth inhibition zones around the antimicrobial discs were measured using a precision digital caliper (Absolute, Mitutoyo, Japan). *E. coli* (Migula) Castellani and Chalmers (ATCC® 25922™) was used as a control. The diameters of the inhibition halos were then measured using a ruler. Antibiotic susceptibility was interpreted according to CLSI guidelines; bacteria were classified as resistant, intermediate, or sensitive [30]. Isolates resistant to ≥ 3 different categories of antibiotics were classified as Multidrug-resistant (MDR), and those resistant to ≥ 6 different categories of antibiotics were classified as Extremely drug-resistant (XDR) [31]. Antibiotics were selected based on their use in treating human infections caused by Gram-negative bacteria and represent different classes of antimicrobial agents available for treating these infections in Mexico [32].

Statistical analysis.

Associations between nominal variables were analyzed using Fisher's exact test and/or chi-squared. Differences in the level of cytotoxicity of DEC strains were determined by one-way analysis of variance, with a p-value ≤0.05 considered statistically significant; IBM® SPSS® Statistics version 20 (New York, USA) was used to perform the statistical analyses, while SigmaPlot version 12 (CA, USA) was used to construct the graphs.

Results

Prevalence of DEC pathotypes in children with diarrhea.

Of the 230 samples collected from children with diarrhea, we analyzed 147; 83 diarrhea samples were excluded, three of which were positive for *Salmonella* or *Shigella flexnerii*, and the rest were not DEC. Sixty-three percent (147/230) of the samples were positive for at least one *E. coli* pathotype, and their distribution in the six health jurisdictions of the state of Oaxaca is shown in Fig. 1.

Figure 1. Geographical locations of sampling sites and pathotypes identified by the municipality of Oaxaca state, southwest Mexico.

Children were divided into five age groups (≤12, 13-24, 25-36, 37-48, and 49-60 months), 46.3% (68/147) were ≤12 months, 18.4% (27/147) 13-24 months, 4.1% (6/147) 25-36 months, 2% (3/147) 37-48 months and 29.3% (43/147) 49-60 months (Table 1). Seven pathotypes were individually identified in children with diarrhea; the most common pathotype found in the samples was aEPEC 51% (75/147), followed by EAEC 10.2% (15/147), in smaller proportions tEPEC (5.4%, 8/147), DAEC (4.8%, 7/147), ETEC (4.1%, 6/147), EIEC (1.4%, 2/147) and EHEC (0.7%, 1/147) (Table 1). In addition, co-infections of two or three DEC were found in the samples, seven co-infections of two pathotypes, and the most common was EAEC+aEPEC 7.5% (11/147), followed by DAEC+aEPEC 4.1% (6/147), the other five combinations (ETEC+aEPEC, EIEC+aEPEC, aEPEC+tEPEC, tEPEC+EIEC and EAEC+DAEC) were found in lower proportion, Table 1. As for the co-infection of three pathotypes, ETEC+aEPEC+EAEC and EIEC+tEPEC+aEPEC were found in one sample each (Table 1).

Table 1. Diarrheagenic *Escherichia coli* (DEC) isolated from children with diarrhea by age group

E. coli pathotypes	Total children n=147 (%)	Age group (months) ≤ 12 (%) n=68 (46.3)	13-24 (%) n=27 (18.4)	25-36 (%) n=6 (4.1)	37-48 (%) n=3 (2)	49-60 (%) n=43 (29.3)	*p*-value
**Single pathotype**							
aEPEC	75 (51.01)	31 (45.6)	17 (63)	3 (50)	1 (33.3)	23 (53.5)	0.169
EAEC	15 (10.2)	11 (16.2) *	2 (7.4)	0 (0.0)	1 (33.3)	1 (2.3)	0.026
tEPEC	8 (5.4)	3 (4.4)	0 (0.0)	0 (0.0)	0 (0.0)	5 (11.6) *	0.033
DAEC	7 (4.8)	6 (8.8) *	1 (3.7)	0 (0.0)	0 (0.0)	0 (0.0)	0.031
ETEC	6 (4.1)	3 (4.4)	1 (3.7)	0 (0.0)	0 (0.0)	2 (4.7)	0.822
EIEC	2 (1.4)	1 (1.5)	0 (0.0)	0 (0.0)	0 (0.0)	1 (2.3)	0.516
EHEC	1 (0.7)	0 (0.0)	1 (3.7)	0 (0.0)	0 (0.0)	0 (0.0)	0 (0.0)
**DEC co-infections**							
EAEC+aEPEC	11 (7.5)	3 (4.4)	1 (3.7)	1 (16.7)	1 (33.3)	5 (11.6)	0.085
DAEC+aEPEC	6 (4.1)	2 (2.9)	2 (7.4)	1 (16.7)	0 (0.0)	1 (2.3)	0.111
ETEC+aEPEC	5 (3.4)	3 (4.4)	1 (3.7)	0 (0.0)	0 (0.0)	1 (2.3)	0.53
EIEC+aEPEC	4 (2.7)	3 (4.4)	0 (0.0)	0 (0.0)	0 (0.0)	1 (2.3)	0.242
aEPEC+tEPEC	3 (2.0)	1 (1.5)	0 (0.0)	1 (16.7)	0 (0.0)	1 (2.3)	0.875
tEPEC+EIEC	1 (0.7)	1 (1.5)	0 (0.0)	0 (0.0)	0 (0.0)	0 (0.0)	0.279
EAEC+DAEC	1 (0.7)	0 (0.0)	0 (0.0)	0 (0.0)	0 (0.0)	1 (2.3)	0.118
ETEC+aEPEC+EAEC	1 (0.7)	0 (0.0)	0 (0.0)	0 (0.0)	0 (0.0)	1 (2.3)	0.118
EIEC+tEPEC+aEPEC	1 (0.7)	0 (0.0)	1 (3.7)	0 (0.0)	0 (0.0)	0 (0.0)	0 (0.0)
**Number Pathotype**							
1	114 (77.5)	55(89.9)	22(81.5)	3(50)	2(66.7)	32(74.4)	0.369
2	31 (21.0)	13(19.1)	4(14.8)	3(50)	1(33.9)	10(23.3)	0.076
3	2 (1.36)	0 (0.0)	1(3.7)	0 (0.0)	0 (0.0)	1(2.3)	0.244

OR: odds ratio, CI: confidence index, *: statistical significance. Chi-square was used to get statistical significance.

Regarding the presence of pathotypes in age groups, aEPEC was the most common pathotype in all age groups (Table 1). However, three pathotypes were associated with age groups, EAEC (16.2%, 11/68) and DAEC (8.8%, 6/68) were associated with children ≤12 months (*p*: 0.026 and *p*: 0.031, respectively), whereas the presence of tEPEC (11.6%, 5/43) was associated with children aged 49-60 months (*p*: 0.033) (Table 1). No associations with any age group were found between other single pathotypes or co-infection (two or three pathotypes).

Association between DEC and clinical characteristics of children with diarrhea.

Several symptoms were recorded in infected children, including fever, vomiting, abdominal pain, and others, and associations with DEC causing diarrhea were sought; individual DEC infection was analyzed as DEC co-infection, individual symptoms, and diarrhea + 1-4 additional symptoms (Table 2). In individual DEC infection, vomiting was the most frequent symptom in children with aEPEC, EAEC and DAEC (52%, 39/75, 66.7%, 10/15 and 71.4%, 5/7, respectively), dehydration, watery diarrhea, bloody diarrhea in tEPEC (50%, 4/8), watery diarrhea in children infected with ETEC (83.3%, 5/6), EIEC and EHEC showed the same prevalence in all symptoms (Table 2). As for diarrhea + 1-4 symptoms, the distribution of symptoms was similar among all DEC pathotypes. Although several symptoms were identified in different DEC, only bloody diarrhea was associated with EIEC (*p*: 0.001), and no association was found between the rest of the individual DEC and symptoms (Table 2).

Table 2. Association between Diarrheagenic *Escherichia coli* (DEC) and clinical symptoms of children with diarrhea.

Clinical Symptoms	Individual DEC infection							*p* value	DEC co-infections									*p* value
	aEPEC n=75 (%)	EAEC n=15 (%)	tEPEC n=8 (%)	ETEC n=6 (%)	DAEC n=7 (%)	EIEC n=2 (%)	EHEC n=1 (%)		EAEC+ aEPEC n=11 (%)	DAEC+ aEPEC n=6 (%)	ETEC+ aEPEC n=5 (%)	EIEC+ aEPEC n=4 (%)	EIEC+ tEPEC n=1 (%)	EAEC+ DAEC n=1 (%)	aEPEC+ tEPEC n=3 (%)	EIEC+ aEPEC+ tEPEC n=1 (%)	ETEC+ EAEC+ aEPEC n=1 (%)	
Fever	27(36.0)	5 (33.0)	0 (0.0)	3(50.0)	3(42.9)	0 (0.0)	1(100)	0.402	3(27.3)	4(66.7)*	0 (0.0)	2(50)	1(100)	0 (0.0)	0 (0.0)	0 (0.0)	0 (0.0)	0.032*
Vomiting	39(52.0)	10(66.7)	3(37.5)	3(50.0)	5(71.4)	1(50.0)	1(100)	0.35	7(63.6)	5(83.3)	2(40)	2(50)	1(100)	1(100)	1(33.3)	0 (0.0)	0 (0.0)	0.158
Abdominal pain	23(30.7)	4(26.7)	1(12.5)	4(66.7)	3(42.9)	0 (0.0)	1(100)	0.057	4(36.4)	4(66.7)	2(40)	3(75)	1(100)	0 (0.0)	1(33.3)	1(100)	0 (0.0)	0.257
Dehydration	38(50.7)	9(60)	4(50.0)	3(50.0)	4(57.1)	1(50.0)	1(100)	0.539	6(54.5)	5(83.3)	3(60)	2(50)	1(100)	0 (0.0)	1(33.3)	0 (0.0)	0 (0.0)	0.117
Watery diarrhea	37(49.3)	9(60)	4(50.0)	5(83.3)	4(57.1)	0 (0.0)	0 (0.0)	0.111	7(63.6)	5(83.3)	4(80)	1(25)	0 (0.0)	1(100)	1(33.3)	0 (0.0)	0 (0.0)	0.158
Mucus diarrhea	34(45.3)	6(40)	4(50.0)	1(16.7)	3(42.9)	1(50.0)	1(100)	0.716	3(27.3)	1(16.7)	1(20)	2(50)	0 (0.0)	0 (0.0)	2(66.7)	1(100)	1(100)	0.199
Bloody diarrhea	4(5.3)	0 (0.0)	0 (0.0)	0 (0.0)	0 (0.0)	1(50.0)	1(100)	0.001*	1(9.1)	0 (0.0)	0 (0.0)	1(25)	1(100)	0 (0.0)	0 (0.0)	0 (0.0)	0 (0.0)	0.237
Only diarrhea	21(28)	2(13.3)	3(37.5)	0 (0.0)	1(14.3)	1(50)	0 (0.0)	0.171	3(27.3)	0 (0.0)	1(20)	1(25)	0 (0.0)	0 (0.0)	1(33.3)	0 (0.0)	1(100)	0.018
Diarrhea + any symptom																		
1	12(16)	4(26.7)	3(37.5)	2(33.3)	2(28.6)	0 (0.0)	0 (0.0)	0.08	1(9.1)	1(16.7)	2(40)*	0 (0.0)	0 (0.0)	1(100)	1(33.3)	1(100)	0 (0.0)	0.002
2	18(24)	4(26.7)	1(12.5)	2(33.3)	0 (0.0)	1(50)	0 (0.0)	0.305	3(27.3)	1(16.7)	1(20)	1(25)	0 (0.0)	0 (0.0)	1(33.3)*	0 (0.0)	0 (0.0)	0.018
3	17(22)	4(26.7)	1(12.5)	1(16.7)	3(42.9)	0 (0.0)	0 (0.0)	0.073	3(27.3)*	1(16.7)	1(20)	1(25)	0 (0.0)	0 (0.0)	0 (0.0)	0 (0.0)	0 (0.0)	0.0001
4	7(9)	1(6.7)	0 (0.0)	1(16.7)	1(14.3)	0 (0.0)	1(100)	0.382	1(9.1)	3(50.0)	0 (0.0)	1(25)	1(100)	0 (0.0)	0 (0.0)	0 (0.0)	0 (0.0)	0

OR: odds ratio, CI: confidence index, *: statistical significance. Chi-square was used to get statistical significance.

Regarding DEC co-infection, watery diarrhea was the most common symptom in EAEC+ aEPEC and ETEC+ aEPEC infected children (63.3%, 7/11 and 80%, 4/5, respectively), fever in DAEC+ aEPEC (66.7%, 4/6), abdominal pain in EIEC+ aEPEC (75%, 3/4), mucoid diarrhea in aEPEC+ tEPEC (66. 7%, 2/3), all other combinations of two or three DEC showed the same prevalence in all symptoms; of all individual symptom totals with DEC co-infection, only fever was associated with DAEC+ aEPEC (*p*: 0.032) compared to all other pathotype combinations (Table 2). As for diarrhea + 1-4 symptoms, there was some association with DEC combinations, diarrhea + 1 symptom was associated with ETEC + aEPEC (40%, 2/5, p: 0.002), diarrhea + 2 symptoms with aEPEC+ tEPEC (33. 3%, 1/3, p: 0.018), diarrhea + 3 symptoms with EAEC+ aEPEC (27.3%, 3/11, p: 0.0001) and diarrhea + 4 symptoms with DAEC+ aEPEC (50%, 3/6, *p*: 0.0001) compared to all other pathotype combinations (Table 2).

Supplementary Virulence Genes (SVG) in DEC Isolated from Children with Diarrhea.

After analyzing the 147 children with diarrhea and possible associations with DEC and some characteristics of the children (age and symptoms), we identified SVG in the DEC strains isolated from the children. Of the 147 diarrheal samples, we isolated 182 DEC, of which aEPEC was the most prevalent DEC with 58.2% (106/182), followed by EAEC 15.3% (28/182), in smaller proportions DAEC (7.1%, 13/182), tEPEC (6.5%, 12/182), ETEC (6.5%, 12/182), EIEC (4.3%, 8/182), and EHEC (0.5%, 1/182) (Table 3).

Table 3. Distribution of SVG in DEC isolated from children with diarrhea.

SVG implied on:	Total DEC n=182 (%)	aEPEC (%) n=106 (58.2)	EAEC (%) n=28 (15.3)	DAEC (%) n=14 (7.9)	tEPEC (%) n=13 (7.1)	ETEC (%) n=12 (6.5)	EIEC (%) n=8 (4.3)	EHEC (%) n=1 (0.5)	*p* value
**Colonization**									
ehaC	166 (91.2)	98 (92.5)	27 (96.4)	13 (92.9)	10 (76.9)	11 (91.7)	6 (75)	1 (100)	0.289
ehaD	112 (61.5)	69 (65.1)	14 (50)	10 (71.4)	6 (46.2)	9 (75)	4 (50)	0 (0.0)	0.321
cah	10 (5.4)	4 (3.8)	0 (0.0)	0 (0)	1 (7.7)	0 (0.0)	0 (0.0)	0 (0.0)	0.257
nleB	8 (4.3)	7 (6.6)	1 (3.6)	0 (0)	0 (0.0)	0 (0.0)	0 (0.0)	1 (100)*	0.001
by number of genes									
0	13 (7.1)	5 (4.7)	1 (3.5)	1 (7.1)	3 (23.0)	1 (8.3)	2 (25.0)*	0 (0.0)	0.044
1	59 (32.4)	34 (32.0)	13 (46.4)	3 (21.4)	4 (30.7)	2 (16.6)	2 (25.0)	1 (100)	0.08
2	100 (54.9)	59 (55.6)	13 (46.4)	10 (71.42)	5 (38.4)	9 (75.0)	4 (50.0)	0 (0.0)	0.14
3	8 (4.9)	6 (5.6)	1 (3.5)	0 (0.0)	1 (7.6)	0 (0.0)	0 (0.0)	0 (0.0)	0.54
4	2 (1.0)	2 (1.8)*	0 (0.0)	0 (0.0)	0 (0.0)	0 (0.0)	0 (0.0)	0 (0.0)	0.001
**Cytotoxicity**									
sat	46 (25.2)	26 (24.5)	6 (21.4)	8 (57.0)*	2 (15.4)	4 (33.3)	0 (0.0)	0 (0.0)	0.004
espC	41 (22.5)	31 (29.2)	0 (0.0)	0 (0.0)	10 (76.9)*	0 (0.0)	0 (0.0)	0 (0.0)	0.001
pet	13 (7.1)	0 (0.0)	13 (46.4)	0 (0.0)	0 (0.0)	0 (0.0)	0 (0.0)	0 (0.0)	-
hlyA	1 (0.5)	0 (0.0)	0 (0.0)	0 (0.0)	0 (0.0)	0 (0.0)	0 (0.0)	1 (100)	-
stx 1	1 (0.5)	0 (0.0)	0 (0.0)	0 (0.0)	0 (0.0)	0 (0.0)	0 (0.0)	1 (100)	-
by number of genes									
0	93 (51.0)	57 (53.7)	11 (39.2)	6 (46.8)	3 (23.0)	8 (66.6)	8 (100)*	0 (0.0)	0.012
1	76 (41.7)	41 (38.6)	15 (53.5)	8 (57.1)	8 (61.5)	4 (33.3)	0 (0.0)	0 (0.0)	0.13
2	12 (6.5)	8 (7.5)	2 (7.1)	0 (0.0)	2 (15.3)	0 (0.0)	0 (0.0)	1 (100)	0.23
3	0 (0.0)	0 (0.0)	0 (0.0)	0 (0.0)	0 (0.0)	0 (0.0)	0 (0.0)	0 (0.0)	-
4	0 (0.0)	0 (0.0)	0 (0.0)	0 (0.0)	0 (0.0)	0 (0.0)	0 (0.0)	0 (0.0)	-
5	0 (0.0)	0 (0.0)	0 (0.0)	0 (0.0)	0 (0.0)	0 (0.0)	0 (0.0)	0 (0.0)	-
**Proteolysis**									
pic	21 (11.5)	14 (13.2)	7 (25)	0 (0.0)	0 (0.0)	0 (0.0)	0 (0.0)	0 (0.0)	0.01
eatA	11 (6.0)	8 (7.5)	1 (3.6)	0 (0.0)	0 (0.0)	2 (16.7)	0 (0.0)	0 (0.0)	0.11
espP	1 (0.5)	0 (0.0)	0 (0.0)	0 (0.0)	0 (0.0)	0 (0.0)	0 (0.0)	1(100)	-
by the number of genes									
0	160 (87.9)	87(82.0)	20 (71.4)	14 (100)	13 (100)	10 (83.3)	8 (100)	0 (0.0)	0.08
1	27 (14.8)	16 (15.0)	8 (28.5)	0 (0.0)	0 (0.0)	2 (16.6)	0 (0.0)	1 (100)	0.02
2	3 (1.6)	3 (2.8)	0 (0.0)	0 (0.0)	0 (0.0)	0 (0.0)	0 (0.0)	0 (0.0)	0.13
3	0 (0.0)	0 (0.0)	0 (0.0)	0 (0.0)	0 (0.0)	0 (0.0)	0 (0.0)	0 (0.0)	-
**Cyclomodulin**									
cnf	18 (9.8)	9 (8.5)	5 (19.9)	1 (7.1)	2 (15.4)	0 (0.0)	1 (12.5)	0 (0.0)	0.12
pks	8 (4.3)	4 (3.8)	3 (10.7)	0 (0.0)	1 (7.7)	0 (0.0)	0 (0.0)	0 (0.0)	0.07
cif	4 (2.1)	2 (1.9)	0 (0.0)	0 (0.0)	1 (7.7)	0 (0.0)	0 (0.0)	1 (100)	0.16
by the number of genes									
0	137 (75.2)	93 (87.7)	22 (78.5)	13 (92.8)	10 (76.9)	12 (100)	7 (87.5)	0 (0.0)	0.15

1	20 (10.9)	11 (10.3)	4 (14.2)	1 (7.14)	2 (15.3)	0 (0.0)	1 (12.5)	1 (100)	0.59
2	10 (5.4)	2 (1.8)	2 (7.1)	0 (0.0)	1 (7.6)	0 (0.0)	0 (0.0)	0 (0.0)	0.25
3	0 (0.0)	0 (0.0)	0 (0.0)	0 (0.0)	0 (0.0)	0 (0.0)	0 (0.0)	0 (0.0)	-
**Iron acquisition**									
ybtS	102 (56.0)	64 (60.4)	15 (53.6)	6 (42.9)	8 (61.5)	6 (50)	3 (37.5)	0 (0.0)	0.67
entB	23 (12.6)	12 (11.3)	5(17.9)	2 (14.3)	2 (15.4)	2 (16.7)	0 (0.0)	0 (0.0)	0.36
by number of genes									
0	73 (40.1)	40 (37.7)	11 (39.2)	7 (50)	4 (30.7)	5 (41.6)	5 (62.7)	1 (100)	0.18
1	93 (51.0)	56 (52.8)	14 (50)	6 (42.8)	8 (61.5)	6 (50)	3 (37.5)	0 (0.0)	0.43
2	16 (8.7)	10 (9.4)	3 (10.7)	1 (7.1)	1 (7.6)	1 (8.3)	0 (0.0)	0 (0.0)	0.69

SVG: supplementary virulence genes, CI: confide index, *: statistical significance. Chi-square was used to get statistical significance.

Seventeen SVG related to colonization (n=4), cytotoxicity (n=5), proteolysis (n=3), cyclomodulins (n=3), and iron acquisition (n=2) were identified in the 182 DEC isolated from children with diarrhea. The distribution of SVG carried by the strains was broad in the DEC; in those related to colonization, of the total DEC, *ehaC* was most prevalent (91.2%, 166/182), followed by *ehaD* (61.5%, 112/182), *cah* (5.4%, 10/182) and *nleB* (4.3%, 8/182); by the number of genes, most strains carried 1 to 2 genes (more than 80%). Regarding pathotype, only the presence of *nleB* was associated with EHEC (100%, 1/1, *p*: 0.001); by the number of genes, the presence of 0 and 4 genes related to SVG colonization were associated with EIEC (25%, 2/8, *p*: 0.044) and aEPEC (1.8%, 2/106, *p*: 0.001), respectively (Table 4).

Regarding cytotoxicity-related genes, *sat* was the most common (25.2%, 46/182), followed by *espC* (22.5%, 41/182), *pet* (7.1%, 13/182), *hlyA*, and *stx1* (0.5%, 1/182), respectively; by number of genes, 51% (93/182) were negative for all genes, while the remaining DEC were positive for 1-2 SVG. *sat*, and e*spC* were associated with DAEC (57%, 8/14, *p*: 0.004) and tEPEC (76.9%, 10/13, *p*: 0.001), respectively, except for EIEC, which was negative for all cytotoxicity-related SVG (*p*: 0.012), the distribution by the number of these SVG was similar in all pathotypes (Table 3). For proteolysis-related SVG, *pic* was the most common (11.5%, 21/182), followed by *eatA* (6%, 11/182) and *espP* (0.5%, 1/182); 87.9% of DEC were negative for these SVG, and the remaining strains were positive for 1-2 genes. EAEC was associated with *pic* (25%, 7/28, *p*: 0.01) and one gene (28.5%, 8/28, *p*: 0.02); the distribution of the remaining SVG in DEC was similar (Table 3).

On the other hand, the presence of cyclomodulins in DEC was limited, *cnf* being the most abundant (9.8%, 18/182), then *pks* (4.3%, 8/182) and *cif* (2.1%, 4/182); 75.2% (137/182) of the strains were negative for any gene, while 16. 3% were positive for 1-2 genes. In this SVG group, no association was found with *E. coli* pathotypes by gene or gene number (Table 3). Similar to the cyclomodulins, in the genes related to iron acquisition, there was no association between these genes (*ybtS*, and *entB*) or the number of these genes with DEC pathotypes (Table 3).

Cytotoxicity of DEC strains with SVG in human cells.

To determine the cytotoxic potential of DEC isolated from children with SVG diarrhea, we tested these strains with HEp-2 cells, and cytotoxic activity was assessed by LDH quantification using a commercial kit. We randomly selected DEC strains positive for some SVG of each identified pathotype, EAEC (n=3), EHEC (n=1), EPEC (n=6), ETEC (n=2), and DAEC (n=2), as a positive damage control, lysis buffer, and E were used. Overall and in their pathotype group, EAEC1 (*ehaC*+, *ehaD*+, *pet*+, and *ytbs*+) and EAEC2 (*ehaC*+, *pet*+, and *ytbs*+) were the most cytotoxic (over 100% and 70%, respectively). Although EHEC harbored seven SVG, it presented a low cytotoxic activity (<40%); it was similar to that of *E. coli* HB101; as for EPEC, EPECa3 strain (*ehaC*+, *sat*+, and *ytbs*+) presented the highest cytotoxic activity (about 60%) compared to the rest of EPEC (Fig. 2). As for ETEC, ETEC1 (*ehaC*+, *ehaD*+, *eatA*+, and *entB*+) presented higher levels of LHD (50%) compared to ETEC2; similar behavior in DAEC, strain DAEC1 (*ehaC*+, *ehaD*+, *sat*+, and *ytbs*+) was the most cytotoxic compared to DAEC2 (Fig. 2).

Figure 2. Cytotoxicity assay of DEC with SVG isolated from children with diarrhea. HEp-2 cells were cultured in supplemented DMEM in a 96-well box at a 30,000 cells/mL density and cultured to monolayer confluence using the incubation conditions described above. Infection was performed at a multiplicity of infection (MOI) of 10 for 12 h in a 5% CO_2 atmosphere at 37°C. DEC pathotypes with different SVG were selected randomly, and *E. coli* HB101 was used as a negative control of cytotoxicity, while buffer lysis was used as a positive control. Cytotoxicity was quantified by LDH release using the CytoTox 96® kit (PROMEGA) according to the manufacturer's instructions. One-way analysis of variance was used to determine the p-value. *: $p < 0.\ 00005$, **: $p < 0.\ 000005$.

Distribution of *E. coli* phylogroups and their association with supplemental virulence genes (SVG).

Different *E. coli* phylogroups (A, B1, B2, and D) were identified by PCR in DEC isolated from children with diarrhea; the most prevalent was A (n=75), followed by B2 (n=48), D (n=38) and B1 (n=21) (Table 4). Regarding pathotypes and phylogroup, aEPEC was the most prevalent in all phylogroups and was associated with phylogroup A (69.3%, 52/75, *p*: 0.011); similarly, DAEC was associated with phylogroup B2 (14.5%, 7/48, p: 0.036) compared to the rest of the phylogroups (Table 4). The remaining pathotypes showed a similar distribution in all phylogroups with no associations.

Table 4. Distribution of phylogroup in *E. coli* isolated from children with diarrhea

E. coli pathotypes	A n=75	B1 n=21	B2 n=48	D n=38	*p*
	n (%)	n (%)	n (%)	n (%)	value
aEPEC	52(69.3)*	12 (57.1)	19 (39.5)	23 (60.5)	0.011
DAEC	2 (2.6)	1 (4.7)	7 (14.5)*	4 (10.5)	0.036
tEPEC	4 (5.3)	2 (9.5)	5 (10.4)	2 (5.2)	0.409
ETEC	4 (5.3)	1 (4.7)	5 (10.4)	2 (5.2)	0.180
EIEC	5 (6.6)	1 (4.7)	1 (2.0)	1 (4.3)	0.187
EAEC	8 (10.6)	4 (19.0)	10 (20.8)	6 (15.7)	0.222
EHEC	0 (0.0)	0 (0.0)	1 (2.0)	0 (0.0)	0
**SVG implied on:**	**n= 28 (%)**	**n=106 (%)**	**n= 13 (%)**	**n= 14 (%)**	***p*-value**
**Colonization**					
ehaC	27 (96.4)	98 (92.5)	10 (76.9)	13 (92.9)	0.289
ehaD	14 (50)	69 (65.1)	6 (46.2)	10 (71.4)	0.321
cah	0 (0.0)	4 (3.8)	1 (7.7)	0 (0.0)	0.257
nleB	1 (3.6)	7 (6.6)	0 (0.0)	0 (0.0)	0
**Cytotoxicity**					
pet	13 (46.4)	0 (0.0)	0 (0.0)	0 (0.0)	0
espC	0 (0.0)	31 (29.2)	10 (76.9)*	0 (0.0)	0.001
sat	6 (21.4)	26 (24.5)	2 (15.4)	8 (57.0)*	0.004
hlyA	0 (0.0)	0 (0.0)	0 (0.0)	0 (0.0)	0
stx 1	0 (0.0)	0 (0.0)	0 (0.0)	0 (0.0)	0
**Proteolysis**					
pic	7 (25) *	14 (13.2)	0 (0.0)	0 (0.0)	0.01
espP	0 (0.0)	0 (0.0)	1 (7.7)	0 (0.0)	0
eatA	1 (3.6)	8 (7.5)	0 (0.0)	0 (0.0)	0.11
**Cyclomodulin**					
pks	3 (10.7)	4 (3.8)	1 (7.7)	0 (0.0)	0.07
cnf	5 (19.9)	9 (8.5)	2 (15.4)	1 (7.1)	0.12
cif	0 (0.0)	2 (1.9)	1 (7.7)	0 (0.0)	0.16
**Iron acquisition**					
entB	5(17.9)	12 (11.3)	2 (15.4)	2 (14.3)	0.36
ybtS	15 (53.6)	64 (60.4)	8 (61.5)	6 (42.9)	0.67

SVG: supplementary virulence genes, CI: confide index, *: statistical significance. Chi-square was used to get statistical significance

Regarding the relationship between *E. coli* phylogroups and SVG, we found some associations Table 2. In genes related to colonization, *ehaC*, and *ehaD* were present in more than 75% and 50% of the phylogroups, respectively, while *cah* was present in phylogroups B1 and B2, as well as *nleB*, which was found in A and B1, despite the distribution of this group of genes in the phylogroups of *E. coli*, no associations were found (Table 4).

Among cytotoxicity-related genes, *pet* was found only in phylogroup A, while espC was associated with B2 (76.9%, 10/13, p: 0.001), also sat was associated with phylogroup D (57%, 8/14, p: 0. 004), *hlyA* and *stx 1* were not found in any phylogroup (Table 4). *pic* was associated with phylogroup A (25%, 7/28, p: 0.01), *espP* was present in phylogroup B2, while *eatA* was present only in A and B1; this in genes related to proteolysis (Table 4). As for cyclomodulin-related genes, *cnf* was found in all phylogroups, *pks* in A, B1, and B2, while *cif* was only in B1 and B2; no associations were found between cyclomodulin and phylogroups (Table 4). Finally, both genes (*entB*, and *ybts*) were found in all *E. coli* phylogroups, but no associations were found (Table 4).

Antimicrobial resistance of *E. coli* strains isolated from children with diarrhea.

Antimicrobial resistance information for DEC strains (n=182) isolated from children with diarrhea (n=147) is shown in Table 5. Overall, more than 50% of the strains were resistant to cefazolin (89.5%), ampicillin (71.4%), streptomycin (64.2%), and trimethoprim (55.4%). By pathotype, EAEC was statistically significantly (*p*: ≤0.05) more resistant to cefotaxime (60.7%), ceftriaxone (28.5%), streptomycin (82.1%), while DAEC was resistant to amoxicillin-clavulanic acid, ampicillin-sulbactam (57. 1% each) and tetracycline (75.8%), ETEC to cefazolin (100%) and imipenem (25%), EIEC to cefazolin (100%) and chloramphenicol (25%); finally, EHEC to cefazolin (100%) (Table 5). By number of antibiotics, 73.9% of DEC were resistant to 1-7 antibiotics, while the rest were resistant to 8-14 antibiotics (Table 5). By category, 79.7% were resistant to any antibiotic, while 3.8% were sensitive to any antibiotic, 43.9% were MDR, and 41.2% were XDR; by pathotype, tEPECs had more

antibiotic-sensitive bacteria (23%, *p*: 0.008), while ETECs had more MDR (75%, *p*: 0.026) and DAECs had higher prevalence of XDR (64.2%, *p*: 0.047) compared to other pathotypes (Table 5).

Table 5. Antibiotic resistance of DEC isolated from children with diarrhea.

Resistance by antibiotic class	Total DEC	*E. coli* pathotypes							*p*-value
		aEPEC (%)	EAEC (%)	DAEC (%)	tEPEC (%)	ETEC (%)	EIEC (%)	EHEC (%)	
	n=182 (%)	n=106 (58.2)	n=28 (15.3)	n=14 (7.6)	n=13 (7.1)	n=12 (6.5)	n= 8 (4.4)	n=1 (0.5)	
**Penicillin**									
Ampicillin	130 (71.4)	81 (76.4)	17 (60.7)	12 (85.7)	8 (61.5)	8 (66.6)	4 (50.0)	0 (0.0)	0.07
**β-Lactamases**									
Amoxicillin- clavulanic acid	61 (33.5)	40 (37.7)	8 (28.5)	8 (57.1)*	4 (30.7)	1 (8.3)	0 (0.0)	0 (0.0)	0.03
Ampicillin sulbactam	47 (28.5)	30 (28.3)	6 (21.4)	8 (57.1)*	3 (23.0)	0 (0.0)	0 (0.0)	0 (0.0)	0.01
**Cephalosporins**									
Cefazolin	163 (89.5)	98 (92.4)	24 (85.7)	12 (85.7)	8 (61.5)	12 (100)*	8 (100)*	1 (100)*	0.012
Cephatoxime	67 (36.8)	33 (31.1)	17 (60.7)*	8 (57.1)	1 (7.6)	7 (58.3)	0 (0.0)	1 (100)	0.004
Ceftriaxone	29 (15.9)	17 (16.0)	8 (28.5)*	0 (0.0)	0 (0.0)	2 (16.6)	2 (25.0)	0 (0.0)	0.047
Ceftazidime	17 (9.3)	13 (12.2)	3 (10.7)	0 (0.0)	0 (0.0)	1 (8.3)	0 (0.0)	0 (0.0)	0.087
**Monobactams**									
Aztreonam	30 (16.4)	20 (18.8)	6 (21.4)	0 (0.0)	0 (0.0)	1 (8.3)	2 (25.0)	1 (100)	0.06
**Carbapenems**									
Imipenem	14 (7.9)	4 (3.7)	4 (14.2)	0 (0.0)	3 (23.0)	3 (25.0)*	0 (0.0)	0 (0.0)	0.019
**Aminoglycosides**									
Kanamycin	31 (17.0)	23 (21.6)	1 (3.5)	1 (7.1)	3 (23.0)	3 (25.0)	0 (0.0)	0 (0.0)	0.334
Streptomycin	117 (64.2)	63 (59.4)	23 (82.1)*	11 (78.5)	7 (53.8)	7 (58.3)	6 (75.0)	0 (0.0)	0.023
**Tetracyclines**									
Tetracycline	89 (48.9)	54 (50.9)	9 (32.1)	11 (78.5)*	6 (46.1)	5 (41.6)	4 (50.0)	0 (0.0)	0.019
**Fluoroquinolones**									
Ciprofloxacin	26 (14.5)	17 (16.0)	6 (21.4)	0 (0.0)	0 (0.0)	3 (25.0)	0 (0.0)	0 (0.0)	0.235
**Quinolones**									
Nalidixic acid	72 (39.5)	47 (44.3)	11 (39.5)	6 (42.8)	1 (7.6)	3 (25.0)	3 (37.5)	1 (100)	0.119
**Folate inhibitor**									
Trimethoprim	101 (55.4)	65 (61.3)	15 (53.5)	11 (78.5)	4 (30.7)	2 (16.6)	4 (50.0)	0 (0.0)	0.06

**Phenicols**									
Chloramphenicol	13 (7.1)	11 (10.3)	0 (0.0)	0 (0.0)	0 (0.0)	0 (0.0)	2 (25.0)*	0 (0.0)	0.044
Resistance by antibiotic number									
1	6 (3.3)	4 (3.7)	1 (3.5)	0 (0.0)	1 (7.6)	0 (0.0)	0 (0.0)	0 (0.0)	0.363
2	18 (9.8)	6 (5.6)	3 (10.7)	1 (7.4)	3 (23.0)	2 (16.6)	3 (37.5)*	0 (0.0)	0.033
3	23 (12.6)	15 (14.1)	3 (10.7)	0 (0.0)	2 (15.3)	3 (25.0)	0 (0.0)	0 (0.0)	0.18
4	24 (13.2)	15 (14.1)	4 (14.8)	0 (0.0)	2 (15.3)	2 (16.6)	0 (0.0)	1 (100)	0.488
5	20 (11.0)	14 13.2)	1 (3.5)	1 (7.4)	1 (7.6)	1 (8.3)	2 (25.0)	0 (0.0)	0.231
6	22 (12.0)	10 (9.4)	5 (17.8)	3 (21.4)	0 (0.0)	2 (16.6)	2 (25.0)	0 (0.0)	0.249
7	22 (12.0)	13 (12.2)	3 (10.7)	4 (28.5)	1 (7.6)	0 (0.0)	1 (12.5)	0 (0.0)	0.071
8	15 (8.2)	11 (10.3)	1 (3.5)	2 (14.2)	0 (0.0)	1 (8.3)	0 (0.0)	0 (0.0)	0.323
9	13 (7.1)	8 (7.5)	3 (10.7)	2 (14.2)	0 (0.0)	0 (0.0)	0 (0.0)	0 (0.0)	0.262
10	5 (2.7)	4 (3.7)	1 (3.5)	0 (0.0)	0 (0.0)	0 (0.0)	0 (0.0)	0 (0.0)	0.304
11	2 (1.1)	2 (1.8)	0 (0.0)	0 (0.0)	0 (0.0)	0 (0.0)	0 (0.0)	0 (0.0)	0.337
12	2 (1.1)	1 (0.9)	1 (3.5)	0 (0.0)	0 (0.0)	0 (0.0)	0 (0.0)	0 (0.0)	0.284
13	3 (1.6)	2 (1.8)	1 (3.5)	0 (0.0)	0 (0.0)	0 (0.0)	0 (0.0)	0 (0.0)	0.396
14	1 0.5)	1 (0.9)	0 (0.0)	0 (0.0)	0 (0.0)	0 (0.0)	0 (0.0)	0 (0.0)	0.582
**Resistance by categories**									
Resistant to any antibiotic	145 (79.7)	105 (99.1)	27 (93.1)	13 (92.8)	0 (0.0)	0 (0.0)	0 (0.0)	0 (0.0)	0
Sensible	7 (3.8)	1 (0.9)	1 (3.5)	1 (7.4)	3 (23.0)*	1 (8.3)	0 (0.0)	0 (0.0)	0.008
MDR	80 (43.9)	44 (41.5)	12 (42.8)	3 (21.4)	8 (61.5)	9 (75.0)*	3 (37.5)	1 (100)	0.026
XDR	75 (41.2)	47 (44.3)	9 (32.1)	9 (64.2)*	7 (53.8)	1 (8.3)	2 (25.0)	0 (0.0)	0.047

MDR= Multidrug-resistant, resistant ≥ 3 different categories of antibiotics; XDR = Extremely drug-resistant≥ 6 different categories of antibiotics. OR: odds ratio, CI: confidence index, *: statistical significance. Chi-square was used to get statistical significance.

Discussion

Diarrhea is a disease that causes many deaths in children, especially in developing countries. In this study, 63% of children with diarrhea from different municipalities of Oaxaca were DEC positive, six pathotypes were identified, and combinations of 2 and 3 pathotypes, with aEPEC being the most prevalent; in addition, associations of single infection or pathotype co-infection with age of children and infant symptomatology were found. Furthermore, associations of SVG related to colonization (*nleB*), cytotoxicity (*sat*, and e*spC*), and proteolysis (*pic*) with different pathotypes were found, as well as associations of SVG related to cytotoxicity (*sat*, and *espC*) and proteolysis (*pic*) with *E. coli* pathotypes. Finally, more than 40% of the DEC were MDR and XDR, with tEPEC and DAEC being the most prevalent.

Diarrhea is common in Mexican children; however, many microorganisms can cause diarrhea. Therefore, the prevalence of DEC in Mexico may vary by region. Patzi-Vargas S et al. (2015) found a high prevalence (56%) of DEC in children with diarrhea in the state of Yucatan, located in southeastern Mexico, data consistent with our findings [26]. In contrast, Angulo-Zamudio et al. (2021) found a prevalence of 18.6% of DEC in children with diarrhea in northwestern Mexico [33]. Similarly, Estrada-García T et al. (2009) reported a 16% prevalence of DEC in children <2 years of age in Mexico City [34].

In this work, aEPEC was the most prevalent isolated pathotype; these data differ from other studies in Mexico [22, 33]. In addition, single infections and co-infections of two or three pathotypes were found in children with diarrhea, a finding also observed in other studies. Rajendran P et al. (2010) found co-infection of more than one pathotype of *E. coli* in 3.8% of all children with diarrhea in South India [35]; unfortunately, the study did not mention pathotype combinations. Nyanga LP et al. (2017) isolated Kenyan children with diarrhea combinations of two (ETEC/EAEC) and three (ETEC/EAEC and EAEC/EPEC/ETEC) *E. coli* pathotypes [36]. In addition, a case was reported in

which up to four *E. coli* pathotypes (STEC, EAEC, ETEC, and aEPEC) were isolated from a 2-year-old child with diarrhea [37].

Some pathotypes were observed to be associated with a specific age of children with diarrhea. Similar to this study, Joffre E. and Iñiguez-Rojas V (2020) found that EAEC was the most common pathotype in 414 children with diarrhea aged 7 to 12 months from Bolivia [38]. Pabst WL et al. (2003) also associated the presence of EAEC with diarrhea in Swiss children aged 0 to 5 years [39]). In contrast to our findings, Ahmed SF et al. (2014) found the highest proportion of EAEC in Egyptian children with diarrhea aged 6-11 years compared to all other age groups [40]. Nguyen RN et al. (2006) identified EPEC as the leading etiologic agent of diarrhea in Australian children aged 16.9 months [41]. Similarly, Chellapandi K et al. (2017) found the highest prevalence of typical and atypical EPEC in diarrhea cases of Indian children aged 0-24 months [42]; all these data differ from our study. DEC infection in infant age groups varies from country to country. Some factors may contribute to DEC infection, such as DEC prevalent in the region, children's immune system, nutritional status, and changes in gut microbiota; in addition, children's feeding (mainly <2 years) is entirely dependent on the mother (through breastfeeding), and maternal hygiene may be crucial for mother-to-child transmission of some DEC [43].

DEC infection in children can cause various symptoms, some associated with specific pathotypes. In this study, bloody diarrhea was associated with EHEC. EHEC has many virulence factors that can cause damage to the host; this bacterium colonizes the intestine, where it causes lesions such as destruction of microvilli, intimate attachment of the bacterium to the intestinal cell, and accumulation of polymerized actin under the site of bacterial attachment to form a pedestal-like structure that hollows out individual bacteria, resulting in bloody diarrhea [4, 44]. For the remaining pathotypes, symptoms such as fever, vomiting, persistent diarrhea, mucoid diarrhea, watery diarrhea, and nausea, among others, have been associated with single EPEC, EPEC, or ETEC infection in various studies [42, 45-47]. However, co-infection with two or three pathotypes may cause more

symptoms in children with diarrhea; Khairy RM et al. (2020) showed that co-infection of EAEC, tEPEC or/and aEPEC presented in higher proportion most of the symptoms presented in children with diarrhea compared to infection by a single DEC. This study agrees with ours as co-infection of different combinations of DEC were the only one associated with diarrhea + other symptoms (1-4) [48].

To cause diarrhea, DEC may need more than their classical virulence factors; in this work, we found associations between some SVG with different pathotypes that could contribute to the development of diarrhea in children in our region. EHEC was associated with *nleB* (related to SVG colonization); others had identified this gene in EHEC [49, 50]. *nleB* is involved not only in the colonization process but also in intestinal cell damage and host mortality; furthermore, this gene has been associated with the ability of EHEC to cause hemolytic uremic syndrome and flare. Apparently, this gene is a linchpin for host initiation, progression, and death, since, in a study testing *nleB* mutant strains in mice, they were unable to cause death compared to wild type [51, 52]. Another SVG (related to cytotoxicity) that we found to be associated with DAEC was *sat*. *sat* is an autotransporter toxin that belongs to the V-type secretion pathway-dependent subfamily of Enterobacteriaceae toxin serine protease autotransporters; this gene has been characterized in *E. coli*; however, it has also been identified in DAECs from children with or without diarrhea, with a higher prevalence in those with diarrhea [53]. sat plays a vital role in the intestinal pathogenesis of DAECs, this gene induces lesions in the binding barrier in Caco-2/TC7 monolayers [54, 55].

The prevalence of *espC* found in diarrhea-producing DAECs is close to 50%, consistent with our results [56, 57]. Another cytotoxicity-related SVG that we found associated with tEPEC was e*spC*. *espC* has been previously described in EPEC, and this gene has multiple functions in EPEC pathogenicity. *espC* in EPEC is an autotransporter not encoded by the locus of enterocyte effacement (LEE); the *espC* protein is released into the interior of cells through the type III secretion system, causing cell rounding and detachment (cytopathic effect); In addition, *espC* has been linked to the

ability of EPEC to cause cell death, inducing both apoptosis and necrosis in epithelial cells [58, 59]. The last SVG (proteolysis-related) associated with aEPEC was *pic*. *pic* is a self-transporting serine protease of Enterobacteriaceae typically found in EAEC; in this pathotype, *pic* exhibits proteolytic activity and contributes to mucus hypersecretion to colonize EAEC. However, in aEPEC, Pic has a different activity; for example, Pic agglutinates erythrocytes, cleaves mucin, and degrades complement system molecules. Moreover, in an in vivo model, Pic contributed to the colonization and mucus production of aEPEC in mice [60].

On the other hand, no association was found between cyclomodulin-related SVG and iron acquisition; however, it is noteworthy that DEC that causes diarrhea also harbor cyclomodulins (*pks*, *cif*, and *cnf*), genotoxins that can alter the DNA of intestinal cells and produce tumors [17]. Apparently, SVGs related to colonization, cytotoxicity, and proteolysis play an important role in the DECs that caused diarrhea in Oaxacan children, besides being the only gene cluster associated with diarrhea cases; in the LDH assay, DEC strains with the highest cytotoxic activity were those with one or more SVGs from this gene cluster (colonization, cytotoxicity, and proteolysis).

In addition, DECs isolated as SVGs in this work were associated with *E. coli* phylogroups. The most prevalent phylogroup in DECs was A, although phylogroup A together with B1 are associated with commensal *E. coli*, DEC strains can also colonize children without causing diarrhea as commensal strains; however, changes in the immune system or microbiota in children may contribute to DECs (as commensals) causing diarrhea [27, 61]. In our study, DECs or SVGs (DAEC, *espC*, and *sat*) were associated with phylogroup B2 or D, which are related to strains of high virulence, extraintestinal *E. coli* that cause urinary tract infections, or strains with high antibiotic resistance [62].

Finally, we analyzed the antimicrobial resistance of diarrhea-producing DEC isolated from Oaxacan children, in which we found high levels of antibiotic resistance. The antimicrobial resistance found in this study is similar to DEC isolated from children with diarrhea in northwestern and central Mexico, with approximately or more than 50% of strains resistant to ampicillin, tetracycline, and

trimethoprim [22, 63]. In addition, more than 40% of the DECs in this study were MDR or XDR, data consistent with other studies [64, 65]. The high antibiotic resistance presented by DECs in developing countries such as Mexico is related to the indiscriminate use of antibiotics, not only in the clinical setting but also in the food industry, agriculture, and livestock. The high antimicrobial resistance of DECs is alarming, and new strategies should be developed to combat this public health problem [66, 67].

This is the first comprehensive work in Mexico that associates DEC with age, symptoms, SVG, phylogroups (the latter two also with each other), and antibiotic resistance. The limitation is the sample size; with larger samples, we could find more associations between DEC and the variables analyzed in this work.

Conclusion

This study provides evidence that children with diarrhea in southeastern Mexico were infected with single or co-infected DECs, aEPEC was the most prevalent pathotype; DECs were associated with age and symptoms of the children. In addition, DEC strains (EHEC, DAEC, tEPEC, and aEPEC) were associated with SVG (*nleB, sat*, e*spC*, and *pic*), as were E. coli pathotypes with SVG. Finally, increased antibiotic resistance was found in DEC strains. DEC research focused on identifying the clinical and molecular characteristics of this pathogen is necessary; rapid identification of pathotypes, virulence factors, and antibiotic resistance could help to choose the best therapy to eradicate DEC infections in children, reducing the number of mortality in this vulnerable population.

Acknowledgments

The authors thank all the staff of the Oaxaca State Public Health Laboratory, for their technical help. We would like to thank Francisco Martínez-Villa for his assistance with the statistical analysis.

Authors' contributions

Gabriela Tapia-Pastrana, **Nidia Leon-Sicairos and Uriel A. Angulo-Zamudio:** Data curation, Writing-Original draft preparation, conceptualization; **Metztli Rojas-Bautista, Pilar Hernández-Pérez:** Visualization, Investigation, Methodology; **Olegario Santiago-Martínez, Lucía C. Gómez-Rodríguez:** Formal analysis; **Víctor M. Terrazas-Luna, Jacobo Montes-Yedra:** Writing-Reviewing and Editing; **Jacobo Montes-Yedra, Alfonso A. Bautista-Avendaño**: Supervision and Reviewing; **Eduardo S. García-López, Nidia Leon-Sicairos:** Software, Validation and Reviewing; **Gabriela Tapia-Pastrana, Uriel A. Angulo-Zamudio, Adrian Canizalez-Roman:** Writing-Original draft preparation, Conceptualization, Project administration, Funding acquisition.

Conflicts of interest

The authors declare that they have no conflicts of interest.

Ethics approval:

The study was approved by the Ethics Committee of the HRAEO project No. HRAEO-CIC-CEI 017/14.

Consent to participate and for publication: All participants were informed about the project and gave verbal and written consent (the parents) to participate.

Data availability

The datasets generated and/or analyzed during the current study are available from the corresponding author on reasonable request.

Funding: This work was supported by grants #25501 and #25101 from the Secretaría de Salud through the Hospital Regional de Alta Especialidad de Oaxaca to G.T.P

References

1. Croxen MA, Law RJ, Scholz R, Keeney KM, Wlodarska M, Finlay BB. Recent advances in understanding enteric pathogenic Escherichia coli. Clin Microbiol Rev. 2013;26(4):822-80. Epub 2013/10/05. doi: 10.1128/cmr.00022-13. PubMed PMID: 24092857; PubMed Central PMCID: PMCPMC3811233.
2. Robins-Browne RM, Holt KE, Ingle DJ, Hocking DM, Yang J, Tauschek M. Are Escherichia coli pathotypes still relevant in the era of whole-genome sequencing? Frontiers in cellular and infection microbiology. 2016;6:141.
3. Trabulsi LR, Keller R, Tardelli Gomes TA. Typical and atypical enteropathogenic Escherichia coli. Emerging infectious diseases. 2002;8(5):508-13. Epub 2002/05/09. doi: 10.3201/eid0805.010385. PubMed PMID: 11996687; PubMed Central PMCID: PMCPMC2732489.
4. Nataro JP, Kaper JB. Diarrheagenic Escherichia coli. Clin Microbiol Rev. 1998;11(1):142-201. Epub 1998/02/11. doi: 10.1128/cmr.11.1.142. PubMed PMID: 9457432; PubMed Central PMCID: PMCPMC121379.
5. Adler B, Sasakawa C, Tobe T, Makino S, Komatsu K, Yoshikawa M. A dual transcriptional activation system for the 230 kb plasmid genes coding for virulence-associated antigens of Shigella flexneri. Molecular microbiology. 1989;3(5):627-35. PubMed PMID: 2474742.
6. Aslani MM, Alikhani MY, Zavari A, Yousefi R, Zamani AR. Characterization of enteroaggregative Escherichia coli (EAEC) clinical isolates and their antibiotic resistance pattern. International journal of infectious diseases : IJID : official publication of the International Society for Infectious Diseases. 2011;15(2):e136-9. doi: 10.1016/j.ijid.2010.10.002. PubMed PMID: 21130676.
7. Jenkins C, Chart H, Willshaw GA, Cheasty T, Smith HR. Genotyping of enteroaggregative Escherichia coli and identification of target genes for the detection of both typical and atypical strains. Diagnostic microbiology and infectious disease. 2006;55(1):13-9. doi: 10.1016/j.diagmicrobio.2005.10.019. PubMed PMID: 16500068.
8. Clements A, Young JC, Constantinou N, Frankel G. Infection strategies of enteric pathogenic Escherichia coli. Gut microbes. 2012;3(2):71-87. doi: 10.4161/gmic.19182. PubMed PMID: 22555463; PubMed Central PMCID: PMC3370951.
9. Piérard D, De Greve H, Haesebrouck F, Mainil J. O157:H7 and O104:H4 Vero/Shiga toxin-producing Escherichia coli outbreaks: respective role of cattle and humans. Veterinary research. 2012;43(1):13. Epub 2012/02/15. doi: 10.1186/1297-9716-43-13. PubMed PMID: 22330148; PubMed Central PMCID: PMCPMC3305544.
10. Patzi-Vargas S, Zaidi MB, Perez-Martinez I, León–Cen M, Michel-Ayala A, Chaussabel D, et al. Diarrheagenic Escherichia coli carrying supplementary virulence genes are an important cause of moderate to severe diarrhoeal disease in Mexico. PLoS neglected tropical diseases. 2015;9(3).
11. Carter MQ, Brandl MT, Kudva IT, Katani R, Moreau MR, Kapur V. Conditional Function of Autoaggregative Protein Cah and Common cah Mutations in Shiga Toxin-Producing Escherichia coli. Applied and environmental microbiology. 2018;84(1). Epub 2017/10/22. doi: 10.1128/aem.01739-17. PubMed PMID: 29054868; PubMed Central PMCID: PMCPMC5734025.
12. Wells TJ, Sherlock O, Rivas L, Mahajan A, Beatson SA, Torpdahl M, et al. EhaA is a novel autotransporter protein of enterohemorrhagic Escherichia coli O157:H7 that contributes to adhesion and biofilm formation. Environ Microbiol. 2008;10(3):589-604. Epub 2008/02/02. doi: 10.1111/j.1462-2920.2007.01479.x. PubMed PMID: 18237301.
13. Melton-Celsa AR. Shiga Toxin (Stx) Classification, Structure, and Function. Microbiology spectrum. 2014;2(4):Ehec-0024-2013. Epub 2014/12/23. doi: 10.1128/microbiolspec.EHEC-0024-2013. PubMed PMID: 25530917; PubMed Central PMCID: PMCPMC4270005.
14. Vieira PCG, Espinoza-Culupú AO, Nepomuceno R, Alves MR, Lebrun I, Elias WP, et al. Secreted autotransporter toxin (Sat) induces cell damage during enteroaggregative Escherichia coli

infection. PLoS One. 2020;15(2):e0228959. Epub 2020/02/23. doi: 10.1371/journal.pone.0228959. PubMed PMID: 32084148; PubMed Central PMCID: PMCPMC7034920.
15. Brunder W, Schmidt H, Karch H. EspP, a novel extracellular serine protease of enterohaemorrhagic Escherichia coli O157: H7 cleaves human coagulation factor V. Molecular microbiology. 1997;24(4):767-78.
16. Restieri C, Garriss G, Locas M-C, Dozois CM. Autotransporter-encoding sequences are phylogenetically distributed among Escherichia coli clinical isolates and reference strains. Appl Environ Microbiol. 2007;73(5):1553-62.
17. Morgan RN, Saleh SE, Farrag HA, Aboulwafa MM. Bacterial cyclomodulins: types and roles in carcinogenesis. Critical reviews in microbiology. 2022;48(1):42-66. Epub 2021/07/16. doi: 10.1080/1040841x.2021.1944052. PubMed PMID: 34265231.
18. Gehring AM, Mori I, Walsh CT. Reconstitution and characterization of the Escherichia coli enterobactin synthetase from EntB, EntE, and EntF. Biochemistry. 1998;37(8):2648-59. Epub 1998/03/28. doi: 10.1021/bi9726584. PubMed PMID: 9485415.
19. Bryce A, Costelloe C, Hawcroft C, Wootton M, Hay AD. Faecal carriage of antibiotic resistant Escherichia coli in asymptomatic children and associations with primary care antibiotic prescribing: a systematic review and meta-analysis. BMC infectious diseases. 2016;16:359. Epub 2016/07/28. doi: 10.1186/s12879-016-1697-6. PubMed PMID: 27456093; PubMed Central PMCID: PMCPMC4960702.
20. Erb A, Stürmer T, Marre R, Brenner H. Prevalence of antibiotic resistance in Escherichia coli: overview of geographical, temporal, and methodological variations. European journal of clinical microbiology & infectious diseases : official publication of the European Society of Clinical Microbiology. 2007;26(2):83-90. Epub 2007/01/20. doi: 10.1007/s10096-006-0248-2. PubMed PMID: 17235554.
21. Kralicek SE, Sitaraman LM, Kuprys PV, Harrington AT, Ramakrishna B, Osman M, et al. Clinical Manifestations and Stool Load of Atypical Enteropathogenic Escherichia coli Infections in United States Children and Adults. Gastroenterology. 2022;163(5):1321-33. Epub 2022/08/11. doi: 10.1053/j.gastro.2022.07.064. PubMed PMID: 35948108; PubMed Central PMCID: PMCPMC9613550.
22. Canizalez-Roman A, Flores-Villaseñor HM, Gonzalez-Nuñez E, Velazquez-Roman J, Vidal JE, Muro-Amador S, et al. Surveillance of Diarrheagenic Escherichia coli Strains Isolated from Diarrhea Cases from Children, Adults and Elderly at Northwest of Mexico. Front Microbiol. 2016;7:1924. Epub 2016/12/15. doi: 10.3389/fmicb.2016.01924. PubMed PMID: 27965648; PubMed Central PMCID: PMCPMC5127799.
23. Dias RCB, Tanabe RHS, Vieira MA, Cergole-Novella MC, Dos Santos LF, Gomes TAT, et al. Analysis of the Virulence Profile and Phenotypic Features of Typical and Atypical Enteroaggregative Escherichia coli (EAEC) Isolated From Diarrheal Patients in Brazil. Front Cell Infect Microbiol. 2020;10:144. Epub 2020/05/12. doi: 10.3389/fcimb.2020.00144. PubMed PMID: 32391284; PubMed Central PMCID: PMCPMC7188757.
24. Kozub-Witkowski E, Krause G, Frankel G, Kramer D, Appel B, Beutin L. Serotypes and virutypes of enteropathogenic and enterohaemorrhagic Escherichia coli strains from stool samples of children with diarrhoea in Germany. Journal of applied microbiology. 2008;104(2):403-10. Epub 2007/09/25. doi: 10.1111/j.1365-2672.2007.03545.x. PubMed PMID: 17887989.
25. Shetty VA, Kumar SH, Shetty AK, Karunasagar I, Karunasagar I. Prevalence and characterization of diarrheagenic Escherichia coli isolated from adults and children in Mangalore, India. Journal of laboratory physicians. 2012;4(1):24-9. Epub 2012/08/28. doi: 10.4103/0974-2727.98666. PubMed PMID: 22923918; PubMed Central PMCID: PMCPMC3425260.
26. Patzi-Vargas S, Zaidi MB, Perez-Martinez I, León-Cen M, Michel-Ayala A, Chaussabel D, et al. Diarrheagenic Escherichia coli carrying supplementary virulence genes are an important cause of moderate to severe diarrhoeal disease in Mexico. PLoS neglected tropical diseases.

2015;9(3):e0003510. Epub 2015/03/05. doi: 10.1371/journal.pntd.0003510. PubMed PMID: 25738580; PubMed Central PMCID: PMCPMC4349884.
27. Angulo-Zamudio UA, Gutierrez-Jimenez J, Monroy-Higuera L, Flores-Villasenor H, Leon-Sicairos N, Velazquez-Roman J, et al. Non-diarrheagenic and diarrheagenic E. coli carrying supplementary virulence genes (SVG) are associated with diarrhea in children from Mexico. Microb Pathog. 2021;157:104994. Epub 2021/05/28. doi: 10.1016/j.micpath.2021.104994. PubMed PMID: 34044054.
28. Canizalez-Roman A, Reina-Reyes JE, Angulo-Zamudio UA, Geminiano-Martinez EE, Flores-Carrillo AF, Garcia-Matus RR, et al. Prevalence of Cyclomodulin-Positive E. coli and Klebsiella spp. Strains in Mexican Patients with Colon Diseases and Antimicrobial Resistance. Pathogens. 2021;11(1). Epub 2022/01/22. doi: 10.3390/pathogens11010014. PubMed PMID: 35055962; PubMed Central PMCID: PMCPMC8779131.
29. Canizalez-Roman A, Gonzalez-Nunez E, Vidal JE, Flores-Villasenor H, Leon-Sicairos N. Prevalence and antibiotic resistance profiles of diarrheagenic Escherichia coli strains isolated from food items in northwestern Mexico. Int J Food Microbiol. 2013;164(1):36-45. Epub 2013/04/17. doi: 10.1016/j.ijfoodmicro.2013.03.020. PubMed PMID: 23587712.
30. White D, Fedorka-Cray P, Chiller C, editors. The national antimicrobial resistance monitoring system (NARMS). NMC-Annual Meeting Proceedings; 2006.
31. Magiorakos AP, Srinivasan A, Carey R, Carmeli Y, Falagas M, Giske C, et al. Multidrug□resistant, extensively drug□resistant and pandrug□resistant bacteria: an international expert proposal for interim standard definitions for acquired resistance. Clinical microbiology and infection. 2012;18(3):268-81.
32. Amabile-Cuevas C. Antibiotic resistance in Mexico: a brief overview of the current status and its causes. Journal of infection in developing countries. 2010;4(3):126-31. PubMed PMID: 20351451.
33. Angulo-Zamudio UA, Gutiérrez-Jiménez J, Monroy-Higuera L, Flores-Villaseñor H, Leon-Sicairos N, Velazquez-Roman J, et al. Non-diarrheagenic and diarrheagenic E. coli carrying supplementary virulence genes (SVG) are associated with diarrhea in children from Mexico. Microb Pathog. 2021;157:104994. Epub 2021/05/28. doi: 10.1016/j.micpath.2021.104994. PubMed PMID: 34044054.
34. Estrada-Garcia T, Lopez-Saucedo C, Thompson-Bonilla R, Abonce M, Lopez-Hernandez D, Santos JI, et al. Association of diarrheagenic Escherichia coli Pathotypes with infection and diarrhea among Mexican children and association of atypical Enteropathogenic E. coli with acute diarrhea. Journal of clinical microbiology. 2009;47(1):93-8. Epub 2008/11/21. doi: 10.1128/jcm.01166-08. PubMed PMID: 19020055; PubMed Central PMCID: PMCPMC2620860.
35. Rajendran P, Ajjampur SS, Chidambaram D, Chandrabose G, Thangaraj B, Sarkar R, et al. Pathotypes of diarrheagenic Escherichia coli in children attending a tertiary care hospital in South India. Diagnostic microbiology and infectious disease. 2010;68(2):117-22. Epub 2010/09/18. doi: 10.1016/j.diagmicrobio.2010.06.003. PubMed PMID: 20846583; PubMed Central PMCID: PMCPMC3918514.
36. Nyanga PL, Onyuka J, Webale MK, Were T, Budambula V. Escherichia coli pathotypes and Shigella sero-groups in diarrheic children in Nairobi city, Kenya. Gastroenterology and hepatology from bed to bench. 2017;10(3):220-8. Epub 2017/11/10. PubMed PMID: 29118939; PubMed Central PMCID: PMCPMC5660273.
37. de Frutos M, Ramiro R, Pereña JI, Sánchez S. Mixed infection of four diarrhoeagenic Escherichia coli pathotypes in a case of travellers' diarrhoea: Caracterisation of the isolates by whole-genome sequencing. Enfermedades infecciosas y microbiologia clinica (English ed). 2020;38(1):39-40. Epub 2019/02/16. doi: 10.1016/j.eimc.2019.01.003. PubMed PMID: 30765107.
38. Joffré E, Iñiguez Rojas V. Molecular Epidemiology of Enteroaggregative Escherichia coli (EAEC) Isolates of Hospitalized Children from Bolivia Reveal High Heterogeneity and Multidrug-

Resistance. Int J Mol Sci. 2020;21(24). Epub 2020/12/19. doi: 10.3390/ijms21249543. PubMed PMID: 33334000; PubMed Central PMCID: PMCPMC7765457.
39. Pabst WL, Altwegg M, Kind C, Mirjanic S, Hardegger D, Nadal D. Prevalence of enteroaggregative Escherichia coli among children with and without diarrhea in Switzerland. Journal of clinical microbiology. 2003;41(6):2289-93. Epub 2003/06/07. doi: 10.1128/jcm.41.6.2289-2293.2003. PubMed PMID: 12791838; PubMed Central PMCID: PMCPMC156476.
40. Ahmed SF, Shaheen HI, Abdel-Messih IA, Mostafa M, Putnam SD, Kamal KA, et al. The epidemiological and clinical characteristics of diarrhea associated with enteropathogenic, enteroaggregative and diffuse-adherent Escherichia coli in Egyptian children. Journal of tropical pediatrics. 2014;60(5):397-400. Epub 2014/08/15. doi: 10.1093/tropej/fmu034. PubMed PMID: 25122630.
41. Nguyen RN, Taylor LS, Tauschek M, Robins-Browne RM. Atypical enteropathogenic Escherichia coli infection and prolonged diarrhea in children. Emerging infectious diseases. 2006;12(4):597-603. Epub 2006/05/18. doi: 10.3201/eid1204.051112. PubMed PMID: 16704807; PubMed Central PMCID: PMCPMC3294699.
42. Chellapandi K, Dutta TK, Sharma I, De Mandal S, Kumar NS, Ralte L. Prevalence of multi drug resistant enteropathogenic and enteroinvasive Escherichia coli isolated from children with and without diarrhea in Northeast Indian population. Annals of clinical microbiology and antimicrobials. 2017;16(1):49. Epub 2017/07/12. doi: 10.1186/s12941-017-0225-x. PubMed PMID: 28693504; PubMed Central PMCID: PMCPMC5504610.
43. Thapar N, Sanderson IR. Diarrhoea in children: an interface between developing and developed countries. Lancet. 2004;363(9409):641-53. Epub 2004/02/28. doi: 10.1016/s0140-6736(04)15599-2. PubMed PMID: 14987892.
44. Nguyen Y, Sperandio V. Enterohemorrhagic E. coli (EHEC) pathogenesis. Front Cell Infect Microbiol. 2012;2:90. Epub 2012/08/25. doi: 10.3389/fcimb.2012.00090. PubMed PMID: 22919681; PubMed Central PMCID: PMCPMC3417627.
45. Okeke IN, Macfarlane-Smith LR, Fletcher JN, Snelling AM. IS3 profiling identifies the enterohaemorrhagic Escherichia coli O-island 62 in a distinct enteroaggregative E. coli lineage. Gut pathogens. 2011;3:4. Epub 2011/04/01. doi: 10.1186/1757-4749-3-4. PubMed PMID: 21450104; PubMed Central PMCID: PMCPMC3079680.
46. Mendez-Arancibia E, Vargas M, Soto S, Ruiz J, Kahigwa E, Schellenberg D, et al. Prevalence of different virulence factors and biofilm production in enteroaggregative Escherichia coli isolates causing diarrhea in children in Ifakara (Tanzania). The American journal of tropical medicine and hygiene. 2008;78(6):985-9. Epub 2008/06/11. PubMed PMID: 18541781.
47. Zhang Y, Tan P, Zhao Y, Ma X. Enterotoxigenic Escherichia coli: intestinal pathogenesis mechanisms and colonization resistance by gut microbiota. Gut microbes. 2022;14(1):2055943. Epub 2022/04/01. doi: 10.1080/19490976.2022.2055943. PubMed PMID: 35358002; PubMed Central PMCID: PMCPMC8973357.
48. Khairy RMM, Fathy ZA, Mahrous DM, Mohamed ES, Abdelrahim SS. Prevalence, phylogeny, and antimicrobial resistance of Escherichia coli pathotypes isolated from children less than 5 years old with community acquired- diarrhea in Upper Egypt. BMC infectious diseases. 2020;20(1):908. Epub 2020/12/02. doi: 10.1186/s12879-020-05664-6. PubMed PMID: 33256619; PubMed Central PMCID: PMCPMC7708180.
49. Bugarel M, Martin A, Fach P, Beutin L. Virulence gene profiling of enterohemorrhagic (EHEC) and enteropathogenic (EPEC) Escherichia coli strains: a basis for molecular risk assessment of typical and atypical EPEC strains. BMC microbiology. 2011;11:142. Epub 2011/06/22. doi: 10.1186/1471-2180-11-142. PubMed PMID: 21689465; PubMed Central PMCID: PMCPMC3133550.
50. Yin X, Zhu J, Feng Y, Chambers JR, Gong J, Gyles CL. Differential gene expression and adherence of Escherichia coli O157:H7 in vitro and in ligated pig intestines. PLoS One.

2011;6(2):e17424. Epub 2011/03/10. doi: 10.1371/journal.pone.0017424. PubMed PMID: 21387009; PubMed Central PMCID: PMCPMC3046156.
51. Wickham ME, Lupp C, Mascarenhas M, Vazquez A, Coombes BK, Brown NF, et al. Bacterial genetic determinants of non-O157 STEC outbreaks and hemolytic-uremic syndrome after infection. The Journal of infectious diseases. 2006;194(6):819-27. Epub 2006/08/31. doi: 10.1086/506620. PubMed PMID: 16941350.
52. Bielaszewska M, Sonntag AK, Schmidt MA, Karch H. Presence of virulence and fitness gene modules of enterohemorrhagic Escherichia coli in atypical enteropathogenic Escherichia coli O26. Microbes and infection. 2007;9(7):891-7. Epub 2007/06/05. doi: 10.1016/j.micinf.2007.03.010. PubMed PMID: 17544311.
53. Taddei CR, Moreno AC, Fernandes Filho A, Montemor LP, Martinez MB. Prevalence of secreted autotransporter toxin gene among diffusely adhering Escherichia coli isolated from stools of children. FEMS microbiology letters. 2003;227(2):249-53. Epub 2003/11/01. doi: 10.1016/s0378-1097(03)00688-8. PubMed PMID: 14592716.
54. Guignot J, Chaplais C, Coconnier-Polter MH, Servin AL. The secreted autotransporter toxin, Sat, functions as a virulence factor in Afa/Dr diffusely adhering Escherichia coli by promoting lesions in tight junction of polarized epithelial cells. Cellular microbiology. 2007;9(1):204-21. Epub 2006/08/15. doi: 10.1111/j.1462-5822.2006.00782.x. PubMed PMID: 16903846.
55. Servin AL. Pathogenesis of human diffusely adhering Escherichia coli expressing Afa/Dr adhesins (Afa/Dr DAEC): current insights and future challenges. Clin Microbiol Rev. 2014;27(4):823-69. Epub 2014/10/04. doi: 10.1128/cmr.00036-14. PubMed PMID: 25278576; PubMed Central PMCID: PMCPMC4187631.
56. Gómez-Duarte OG, Arzuza O, Urbina D, Bai J, Guerra J, Montes O, et al. Detection of Escherichia coli enteropathogens by multiplex polymerase chain reaction from children's diarrheal stools in two Caribbean-Colombian cities. Foodborne pathogens and disease. 2010;7(2):199-206. Epub 2009/10/21. doi: 10.1089/fpd.2009.0355. PubMed PMID: 19839760; PubMed Central PMCID: PMCPMC2904504.
57. Mansan-Almeida R, Pereira AL, Giugliano LG. Diffusely adherent Escherichia coli strains isolated from children and adults constitute two different populations. BMC microbiology. 2013;13:22. Epub 2013/02/05. doi: 10.1186/1471-2180-13-22. PubMed PMID: 23374248; PubMed Central PMCID: PMCPMC3577467.
58. Navarro-Garcia F, Serapio-Palacios A, Vidal JE, Salazar MI, Tapia-Pastrana G. EspC promotes epithelial cell detachment by enteropathogenic Escherichia coli via sequential cleavages of a cytoskeletal protein and then focal adhesion proteins. Infect Immun. 2014;82(6):2255-65. Epub 2014/03/20. doi: 10.1128/iai.01386-13. PubMed PMID: 24643541; PubMed Central PMCID: PMCPMC4019189.
59. Serapio-Palacios A, Navarro-Garcia F. EspC, an Autotransporter Protein Secreted by Enteropathogenic Escherichia coli, Causes Apoptosis and Necrosis through Caspase and Calpain Activation, Including Direct Procaspase-3 Cleavage. mBio. 2016;7(3). Epub 2016/06/23. doi: 10.1128/mBio.00479-16. PubMed PMID: 27329750; PubMed Central PMCID: PMCPMC4916375.
60. Abreu AG, Abe CM, Nunes KO, Moraes CT, Chavez-Dueñas L, Navarro-Garcia F, et al. The serine protease Pic as a virulence factor of atypical enteropathogenic Escherichia coli. Gut microbes. 2016;7(2):115-25. Epub 2016/03/11. doi: 10.1080/19490976.2015.1136775. PubMed PMID: 26963626; PubMed Central PMCID: PMCPMC4856457.
61. Duriez P, Clermont O, Bonacorsi S, Bingen E, Chaventré A, Elion J, et al. Commensal Escherichia coli isolates are phylogenetically distributed among geographically distinct human populations. Microbiology (Reading). 2001;147(Pt 6):1671-6. Epub 2001/06/08. doi: 10.1099/00221287-147-6-1671. PubMed PMID: 11390698.
62. Johnson JR, Kuskowski MA, Owens K, Gajewski A, Winokur PL. Phylogenetic origin and virulence genotype in relation to resistance to fluoroquinolones and/or extended-spectrum cephalosporins and cephamycins among Escherichia coli isolates from animls and humans. The

Journal of infectious diseases. 2003;188(5):759-68. Epub 2003/08/23. doi: 10.1086/377455. PubMed PMID: 12934193.
63. Estrada-García T, Cerna JF, Paheco-Gil L, Velázquez RF, Ochoa TJ, Torres J, et al. Drug-resistant diarrheogenic Escherichia coli, Mexico. Emerging infectious diseases. 2005;11(8):1306-8. Epub 2005/08/17. doi: 10.3201/eid1108.050192. PubMed PMID: 16102327; PubMed Central PMCID: PMCPMC3320511.
64. Dela H, Egyir B, Majekodunmi AO, Behene E, Yeboah C, Ackah D, et al. Diarrhoeagenic E. coli occurrence and antimicrobial resistance of Extended Spectrum Beta-Lactamases isolated from diarrhoea patients attending health facilities in Accra, Ghana. PLoS One. 2022;17(5):e0268991. Epub 2022/05/27. doi: 10.1371/journal.pone.0268991. PubMed PMID: 35617316; PubMed Central PMCID: PMCPMC9135277.
65. Yandag M, Tsend-Ayush A, Gunregjav N, Erdenebayar O, Byambadorj B, Juniichiro N, et al. Detection and antibiotic resistance of diarrheagenic Escherichia coli from patients with diarrhea in Ulaanbaatar, Mongolia. J Infect Dev Ctries. 2023;17(2):202-9. Epub 2023/03/11. doi: 10.3855/jidc.17256. PubMed PMID: 36897900.
66. Marshall BM, Levy SB. Food animals and antimicrobials: impacts on human health. Clin Microbiol Rev. 2011;24(4):718-33. Epub 2011/10/07. doi: 10.1128/cmr.00002-11. PubMed PMID: 21976606; PubMed Central PMCID: PMCPMC3194830.
67. Manyi-Loh C, Mamphweli S, Meyer E, Okoh A. Antibiotic Use in Agriculture and Its Consequential Resistance in Environmental Sources: Potential Public Health Implications. Molecules (Basel, Switzerland). 2018;23(4). Epub 2018/03/31. doi: 10.3390/molecules23040795. PubMed PMID: 29601469; PubMed Central PMCID: PMCPMC6017557.

Figure 1

Click here to access/download;Figure;Figure 1.tif

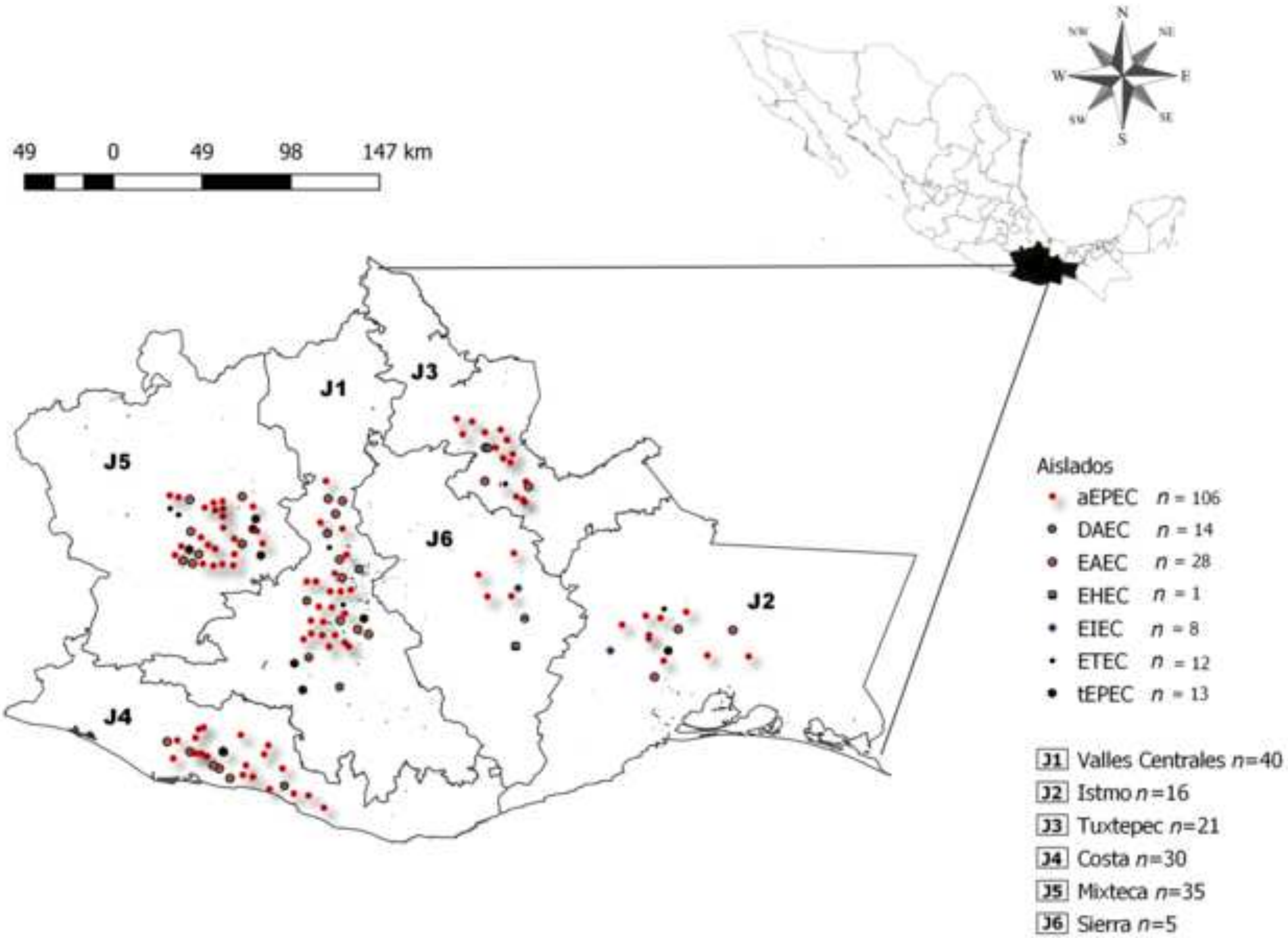

Figure 2

Click here to access/download;Figure;Figure 2.tif

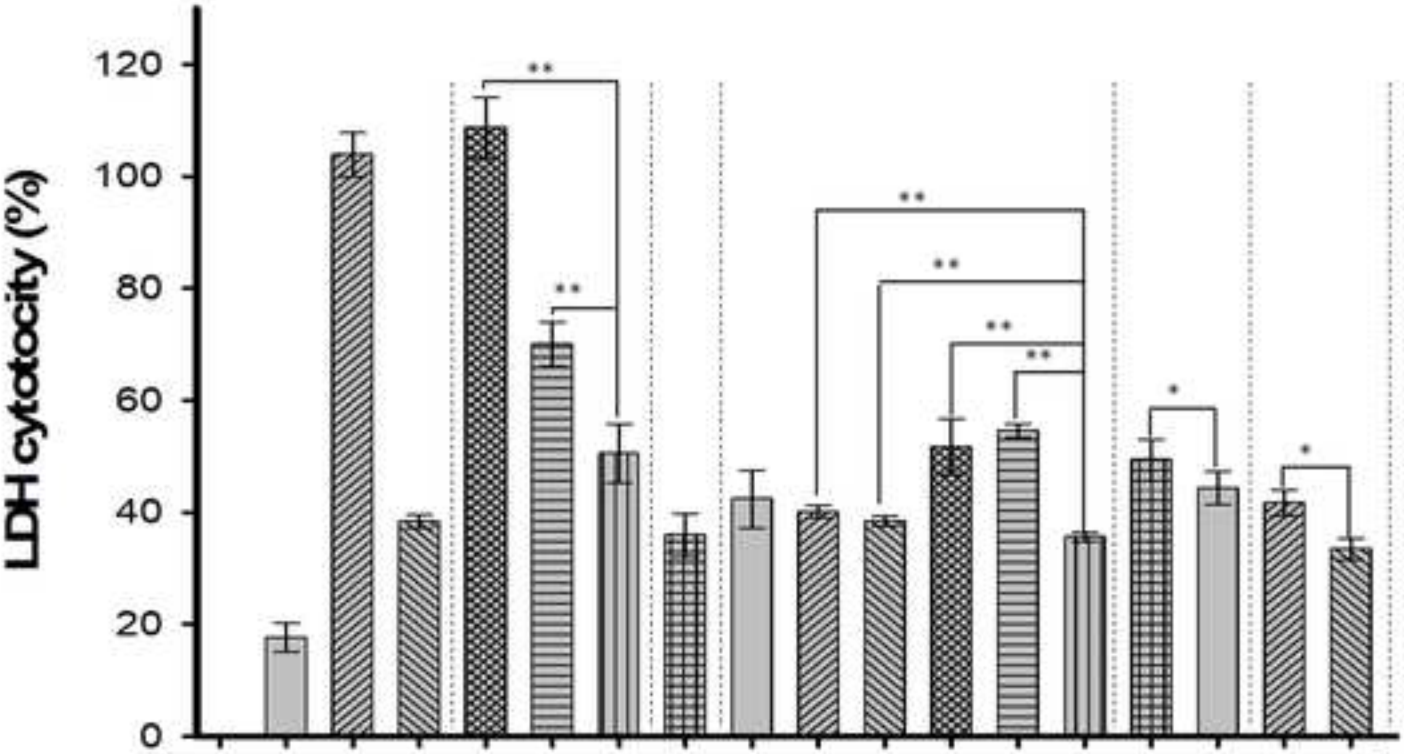

SVG related to	MOCK	Lysis control	HB101	EAEC1	EAEC2	EAEC3	EHEC1	EPECt1	EPECt2	EPECa1	EPECa2	EPECa3	EPECa4	ETEC1	ETEC2	DAEC1	DAEC2
**Colonization**																	
ehaC				+	+	+	+		+	+	+	+	+	+	+	+	+
ehaD				+		+	+			+	+		+	+	+	+	+
cah													+				
nleB							+	+			+						
**Cytotoxicity**																	
sat									+	+		+				+	
espC								+	+	+							
pet				+	+												
hlyA							+										
stx1							+										
**Proteolysis**																	
pic				+							+	+					
eatA														+			
espP							+										
**Cyclomodulin**																	
cif									+		+						
pks						+											
**Iron acquisition**																	
ytbs				+	+					+		+	+			+	
entB						+	+							+			

Click here to access/download
Supporting Information
Supplementary Table 1 PRIMERS MOG GTP (1).docx
